# Towards the Human Colorectal Cancer Microbiome

**DOI:** 10.1371/journal.pone.0020447

**Published:** 2011-05-24

**Authors:** Julian R. Marchesi, Bas E. Dutilh, Neil Hall, Wilbert H. M. Peters, Rian Roelofs, Annemarie Boleij, Harold Tjalsma

**Affiliations:** 1 School of Biosciences, Cardiff University, Cardiff, United Kingdom; 2 Centre for Molecular and Biomolecular Informatics, Nijmegen Centre for Molecular Life Sciences, Radboud University Nijmegen Medical Centre, Nijmegen, The Netherlands; 3 Departments of Computer Science and Biology, San Diego State University, San Diego, California, United States of America; 4 Centre for Genomic Research, School of Biological Sciences, University of Liverpool, Liverpool, United Kingdom; 5 Department of Gastroenterology, Nijmegen Institute for Infection, Inflammation and Immunity (N4i) & Radboud University Centre for Oncology (RUCO) of the Radboud University Nijmegen Medical Centre, Nijmegen, The Netherlands; 6 Department of Laboratory Medicine, Nijmegen Institute for Infection, Inflammation and Immunity (N4i) & Radboud University Centre for Oncology (RUCO) of the Radboud University Nijmegen Medical Centre, Nijmegen, The Netherlands; University of Hyderabad, India

## Abstract

Multiple factors drive the progression from healthy mucosa towards sporadic colorectal carcinomas and accumulating evidence associates intestinal bacteria with disease initiation and progression. Therefore, the aim of this study was to provide a first high-resolution map of colonic dysbiosis that is associated with human colorectal cancer (CRC). To this purpose, the microbiomes colonizing colon tumor tissue and adjacent non-malignant mucosa were compared by deep rRNA sequencing. The results revealed striking differences in microbial colonization patterns between these two sites. Although inter-individual colonization in CRC patients was variable, tumors consistently formed a niche for *Coriobacteria* and other proposed probiotic bacterial species, while potentially pathogenic *Enterobacteria* were underrepresented in tumor tissue. As the intestinal microbiota is generally stable during adult life, these findings suggest that CRC-associated physiological and metabolic changes recruit tumor-foraging commensal-like bacteria. These microbes thus have an apparent competitive advantage in the tumor microenvironment and thereby seem to replace pathogenic bacteria that may be implicated in CRC etiology. This first glimpse of the CRC microbiome provides an important step towards full understanding of the dynamic interplay between intestinal microbial ecology and sporadic CRC, which may provide important leads towards novel microbiome-related diagnostic tools and therapeutic interventions.

## Introduction

The human intestinal tract contains about 10^14^ bacteria, comprising ∼10^3^ species, which are essential for digestion of food, the control of intestinal epithelial homeostasis, intestinal development and human health [1]. Conversely, a large body of evidence supports a relationship between infective agents and human cancers [2] and suggests that certain mucosa-associated bacterial species play an important role in the pathogenesis of colorectal cancer (CRC; [3], [4], [5]. Moreover, clinical associations between bacterial infection and CRC have been described for many decades, the most prominent of which concern infections with *Streptococcus bovis* [6], [7] and *Clostridium septicum* [8]. However, the co-incidence of these infections with CRC is very low (<1%) since such low-grade opportunistic pathogens can only become clinically manifest in compromised patients. Correspondingly, serological data have shown an increased exposure to *S. bovis* antigens in early stage CRC patients without clinical signs of bacterial infection [9]. Based on this, it has been suggested that specific gut bacteria have a competitive advantage in the CRC microenvironment, whereas opportunistic infections remain repressed by the active immune system in the majority of patients.

Recent publications have provided mechanistic evidence for the involvement of gut bacteria in the development of CRC, which comprises i), production of DNA damaging superoxide radicals, ii) production of genotoxins, iii) T helper cell-dependent induction of cell proliferation, iv) Toll-like receptor mediated induction of pro-carcinogenic pathways [10]–[14]. Despite this vast body of circumstantial evidence, however, no clinical data have thus far been available to directly show distinct bacterial colonization patterns in CRC patients. In fact, the molecular nature of the complex intestinal community was largely unexplored prior to the moment that Eckburg and coworkers [15] revealed the presence of ∼400 bacterial species by sequencing prokaryotic ribosomal RNA gene sequences from multiple colonic mucosal sites and feces of healthy subjects. Further investigations revealed high intra-individual variation of intestinal microbiomes in the human population, whereas the microbial colonization of the mucosa within adult individuals is relatively stable throughout the colon [16]–[18]. Based on the latter observations we hypothesized that the in-depth analysis of a relatively small number of paired on/off-tumour tissue samples from CRC patients could disclose bacterial species that might be implicated in CRC etiology. To achieve this goal, we used deep pyrosequencing of bacterial rRNA to compare CRC tumor microbiomes to that of adjacent non-malignant mucosa across six patients. The data provided the first high-resolution image of the human CRC microbiome and showed that CRC is associated with quite dramatic shifts in the adherent intestinal microbiota.

## Materials and Methods

### Patient Material

Six patients (labeled A–F, Table 1) underwent resections for primary colon adenocarcinoma at the Radboud University Nijmegen Medical Centre. After resection, the colonic specimens were extensively rinsed with sterile water after which the specimens were examined by an oncological pathologist. Disease was staged according to the Tumor-Node-Metastasis (TNM) classification [19]. From each colonic specimen, biopsies were taken from the tumor site (“on-tumor”, A_on_–F_on_) and from adjacent non-malignant tissue (“off-tumor”, A_off_–F_off_) on the luminal side of the colonic wall (distance about 5–10 cm). Tissue specimens were disrupted by mechanical shearing after which total DNA was extracted using the AllPrep DNA/RNA kit (Qiagen). All samples were stored at −80°C until use.

**Table 1:** Patient characteristics.

			Colon Tumor	
Patient	Gender	Age	Stage^1^	Localisation
A	m	67	T_2_N_0_M_0_	sigmoid
B	m	61	T_2_N_0_M_0_	rectum/sigmoid
C	m	49	T_3_N_1_M_0_	sigmoid
D	m	71	T_2_N_0_M_0_	rectum
E	m	67	T_4_N_0_M_0_	cecum
F	f	66	T_2_N_0_M_0_	rectum

1 T, tumor growth into the wall of the intestine; N, spread to nearby lymph nodes; M, metastases in other organs; numbers 0–4 indicate increasing severity.

### Ethics Statement

Research was conducted according to the principles expressed in the Declaration of Helsinki. The study was approved by the by the Medical Ethical Committee of the district Arnhem-Nijmegen (The Netherlands); patients provided written informed consent for the collection of samples and subsequent analysis when required.

### Denaturing Gradient Gel Electrophoresis (DGGE) and Ribosomal Intergenic Spacer Analysis (RISA)

**Figure 1:**
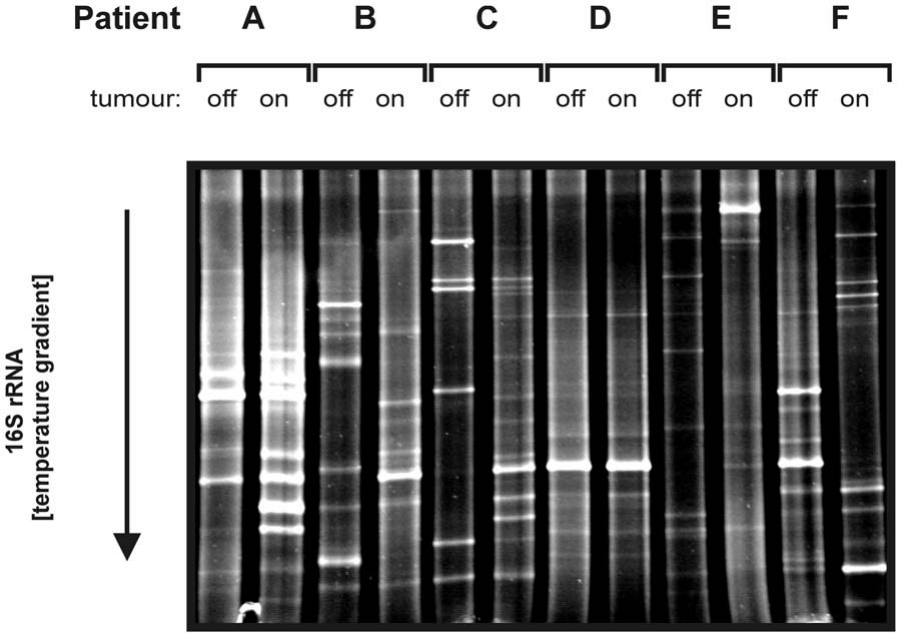
DGGE Fingerprinting of CRC Tissue and Non-malignant Adjacent Mucosa. An internal fragment (∼450 bps) of the bacterial 16S rRNA gene was amplified from colon tissue-extracted DNA by a broad-range PCR approach after which these amplicon mixtures were applied to DGGE. Patient characteristics can be found in Table 1; *off*, non-malignant tissue; *on*, tumor tissue.

Using total DNA from the 12 colonic biopsies as a template, bacterial 16S rRNA genes were amplified by a nested approach [20] using the primers pairs 27f/1492r [21] and L1401r/968f-GC [22], [23] in two subsequent PCR reactions (Table S1). DGGE was performed on the resulting PCR mixture as described previously [24]. It should be noted that visible bands (see Figure 1) represent bacterial species that have an abundance of at least 1–10% of the total community, whereas low abundant species will not result in a detectable bands by this approach. To confirm DGGE data, bacterial ribosomal intergenic spacer regions were amplified with primers 1406f and 23Sr using the same DNA as template (Table S1; [24]). RISA was performed as described previously [23].

### FLX 454 titanium pyrosequencing

In the second step of the nested PCR approach, we amplified the V1–V3 region of the bacterial 16S rRNA gene using primer pairs tagged with 12 distinct **M**etagenome **ID**entification (MID) tags (Table S1). 454 sequencing was performed at the University of Liverpool's Advanced Genomics Facility. Sequences are available on request.<

### Read processing and community diversity

All partial 16S rRNA gene sequences were processed initially using the Pyro-pipeline at the Ribosomal database project (RDP, [25]; Release 10) to trim and remove primers from the partial ribotags and to limit sequences to >400 bp and < = 500 bp, sequences were processed using the pre.cluster command which minimizes errors introduced by the pyrosequencing platform [26]. This step provided the datasets for analysis (Table S2) with the read length histograms shown in Figure S2*A*. The data from all the samples was processed using MOTHUR [27] to generate indices of diversity, rarefaction curves (Figure S2*B*) and to undertake the Libshuff analysis of sample similarity. MOTHUR was run using the computational facilities of the Advanced Research Computing @ Cardiff (ARCCA) Division, Cardiff University. Comparisons of the libraries from an individual was performed using the RDP's Library compare tool. Analysis of the ribotags was also performed using MEGAN [28] for which the input was the csv output from the RDP's classifier pipeline (using default settings and a confidence level of 50%). The comparison tool was selected and reads normalized between samples and Bonferroni correction used to highlight differences between samples. An alignment independent analysis of the date was also undertaken using 5-mers and frequency landscape distribution (fLAND) analysis [29]–[31]. The generation of the 5-mers was performed using a bespoke PERL script (written by BED; available on request) and PCA analysis was undertaken in MATLAB on ARCCA, the fLAND analysis was performed using the software fLAND.

### Consistency analysis

Biases in microbiota between the on-tumor and off-tumor samples across patients were summarized in order to identify taxa which were either consistently enriched or consistently depleted in the two niches. All pyrosequencing reads were first mapped to the SILVA comprehensive database of aligned, quality checked 16S/18S rRNA sequences >300 nt (version SSUParc_100; [32]) using BLAT v34 with default parameters and cutoffs [33]. We assumed that each read was derived from a different micro-organism and that the sampling of reads represented the taxonomic distribution within the intestinal microbiota. For each sample, every read was assigned to its most similar sequence in the SILVA database and a summary of the taxonomic annotations of the detected database sequences was generated. Each taxonomic clade was assessed to determine whether it showed a higher fraction of reads in off-tumor or on-tumor samples for every patient, and a consistency score was calculated by counting “+1” if the clade was higher off-tumor, “−1” if the clade was higher on-tumor, and “0” if the fraction of reads on- and off-tumor was identical (e.g. if the clade was not measured in this patient). Finally, these scores were summed, yielding an overall consistency score between −6 and +6 that reflects how consistently the clade was enriched or depleted across all patients. Note that each sequence in the SILVA database has two taxonomic annotations, i.e. EMBL and RDP.

## Results

### DGGE fingerprinting

As a first exploration, profiles of bacterial 16S rRNA genes were generated using DGGE for tumor and matching adjacent non-malignant (off-tumor) mucosa from 6 CRC patients (Table 1). As shown in Figure 1, the microbial communities of tumor tissue and adjacent “off-tumor” mucosa were strikingly different and similar results were obtained when RISA fingerprinting was applied to the same samples (Figure S1). Notably, this result sharply contrasts with the previous observations that the colonic mucosal microbiota is almost identical at adjacent sites in healthy subjects [15], [16], [34]–[36]. Inspired by this striking observation, we next aimed for a microbiome sequencing approach to map the microbiome changes at a high resolution rather than sequencing of individual distinguishing bands that only represent the abundant species in a certain sample.

### FLX 454 titanium pyrosequencing

**Figure 2:**
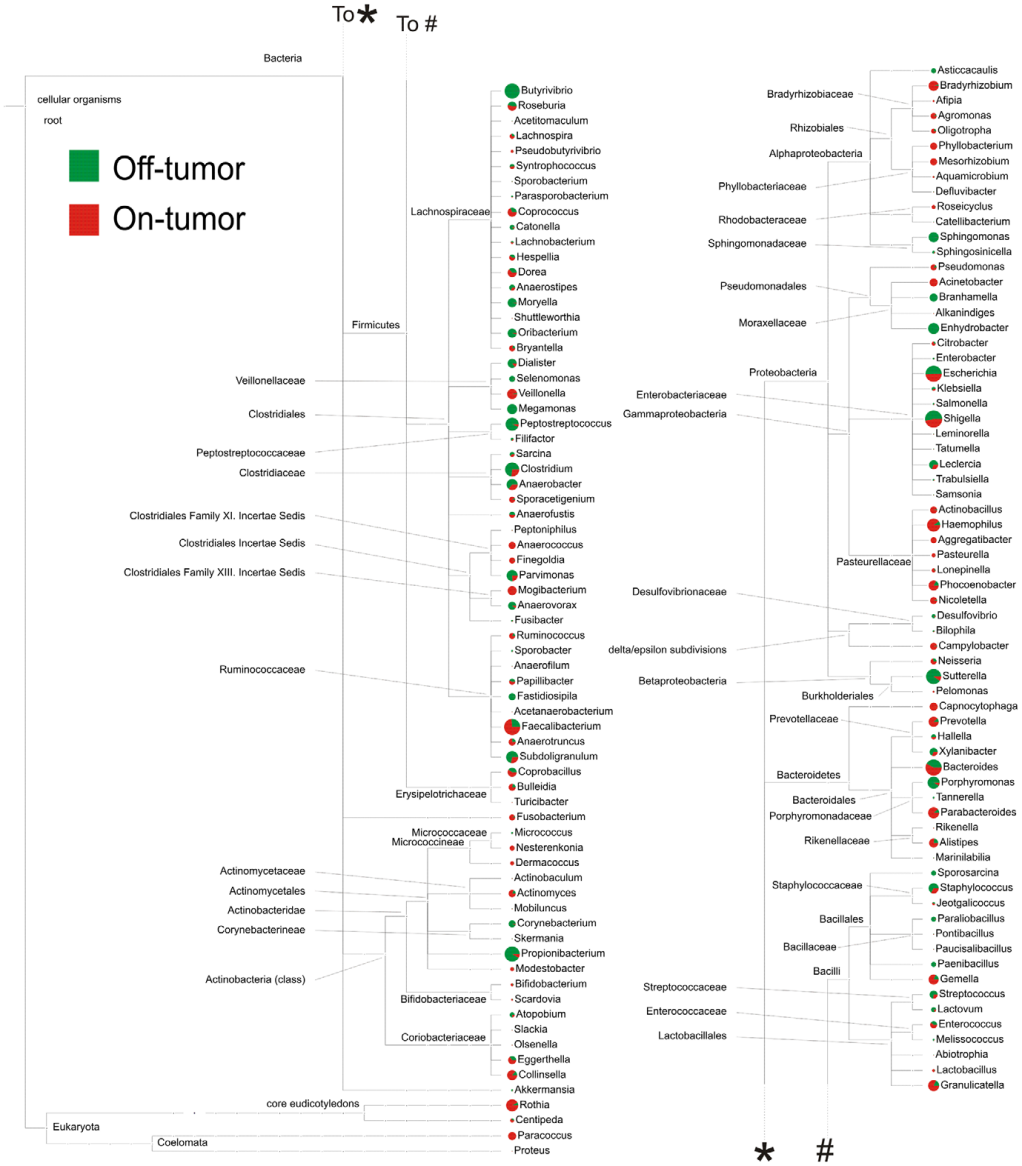
Phylogenetic Analysis of Altered Microbiomes. The 454 sequencing data were normalized in MEGAN (Huson et al. 2009) and parsed through the RDP pyropipeline classifier tool (Cole et al. 2009) to generate a csv file of taxonomic abundance. This file was used as input for MEGAN to visualize in which families differences between non-malignant tissue (*off*-tumor) and CRC tissue (*on*-tumor) communities are present. A high-resolution image of this Figure for “zoom-in” purposes can be downloaded from Figure S2*E*.

To define the colon tumor microbiome at a deep level, we amplified and sequenced the V1–V3 region of the bacterial 16S rRNA genes (Table S1), which resulted in a total of 193,880 ribotags of length 401–500 bp. The data showed high coverage values (>88%) and rarefaction curves indicated satisfactory sampling of the communities at 90% identity (Figure S2*B*; Table S2). Libshuff analysis indicated that all on- and off-tumor communities were significantly different (p<0.0001) from each other (Table S3). Importantly, both alignment-dependent and independent methods supported the observation of these altered tumor microbiomes (Figure 2, Figure S2 *C* and *D*; Table S3). Moreover, while DGGE and RISA only showed minor differences for patient D, subtle, but significant, differences could be identified by the deep sequencing approach. This clearly exemplifies the superior resolution that can be obtained with the latter technology. The data showed a general tendency of more Bacteroidetes and less Firmicutes from in tumor tissue compared to matching off-tumor mucosa (Figure S3). However, as could be expected the observed microbiome shifts showed a high level of variability among patients (Figure S3A–F) and in certain cases were even contrary to the general tendency (Figure S3*G*). Although *S. bovis* or *C. septicum* infections have a known clinical association with CRC, only very few sequences mapped to rRNA of these two species and no dependable colonization of CRC tissue was observed. This could be explained by the fact that such opportunistic pathogens are predominantly present in the transient adenoma stage of CRC [37].

### Consistency analysis

**Figure 3:**
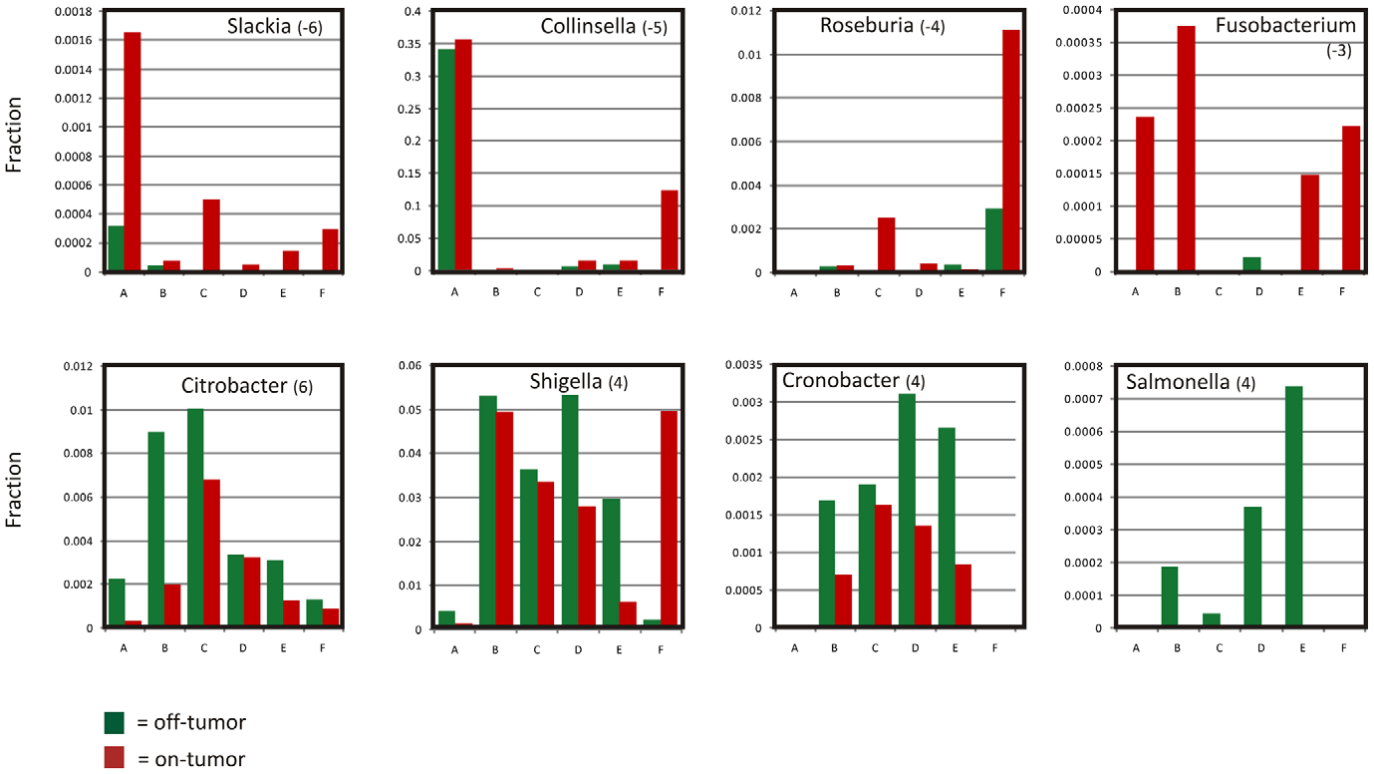
Consistent Biases in Microbiota CRC Tissue and Non-malignant Adjacent Mucosa. Relative distribution of selected CRC over/under represented taxa was calculated as the fraction annotated sequences of the total number of reads in that specific sample. Consistency score for the indicated taxon (see Table 2) is given between brackets and reflects how consistently clades were enriched across patients A–F; green bars indicate the fraction in *off*-tumor and red bars indicate fraction of this taxon *on*-tumor.

To pinpoint the most imperative microbiome changes during formation of CRC, consistency across the patients was calculated for each taxon individually by giving it a score of “−1” if the normalized number of sequence reads of that taxon in the tumor tissue was higher than in matching off-tumor mucosa, “+1” if the taxon was more abundant in healthy mucosa and “0” if it was not detected at all in that patient. Summing these scores across patients resulted in a consistency score from −6 to +6 for each taxon (Tables 2 and S4, Figure S4*A* and *B*). It should be noted that some sequences are better annotated by EMBL than by RDP, and *vice versa* (see Figure S4C), so we report both rather than preferentially trusting either of these taxonomic annotations. This approach showed that CRC tissue was consistently associated with overrepresentation of the subclass of *Coriobacteridae*, especially the genera *Slackia* and *Collinsella*, which can be regarded as gut commensals. On the other hand, members of the *Enterobacteriaceae*, such as *Citrobacter*, *Shigella*, *Cronobacter*, *Kluyvera*, *Serratia* and *Salmonella* spp. (scores between +4 and +6; Table 2; Figure S4*A* and *B*) were underrepresented in CRC tissue. Although these findings were consistent, the relative abundance of these taxa differed considerably between on and off tumor mucosa from the investigated patients as depicted in Figure 3.

**Table 2:** Consistent CRC-associated Microbiome Shifts^1^.

	consistency score		
Terminal Clade	EMBL	RDP	Bacterial order
*Overrepresented in CRC tissue*			
*Slackia*	.	−6	*Coriobacteriales*
*Collinsella*	.	−5	*Coriobacteriales*
*Eubacterium*;environmental samples	−5	.	*Clostridiales*
*Coriobacterium*;environmental samples	−4	.	*Coriobacteriales*
*Roseburia*	−4	.	*Clostridiales*
*Clostridiaceae*;environmental samples	−4	.	*Coriobacteriales*
*Fusobacterium*	−3	−3	*Fusobacteriales*
unclassified_*Coriobacteriaceae*	.	−3	*Coriobacteriales*
unclassified_*Peptostreptococcaceae*	.	−3	*Clostridiales*
*Erysipelotrichaceae* Incertae Sedis	.	−3	*Erysipelotrichales*
*Fusobacteria*;environmental samples	−3	.	*Fusobacteriales*
*Faecalibacterium*;environmental samples	−3	.	*Clostridiales*
*Bacteroidales*;environmental samples	−3	.	*Bacteroidales*
unclassified_Firmicutes	-	−3	*N/A*
*Underrepresented in CRC tissue*			
*Citrobacter*	6	4	*Enterobacteriales*
*Shigella*	4	4	*Enterobacteriales*
*Cronobacter*	4	.	*Enterobacteriales*
*Kluyvera*	4	.	*Enterobacteriales*
*Serratia*	4	.	*Enterobacteriales*
*Salmonella enterica* subsp. enterica serovar Saintpaul	4	.	*Enterobacteriales*
*Eubacteriaceae*; environmental samples	4	.	*Clostridiales*
*Anaerovorax*	.	4	*Clostridiales*
unclassified_Ruminococcaceae	.	4	*Clostridiales*
unclassified_Bacteria	.	4	N/A
*Microbacterium*	3	.	*Actinomycetales*
*Salmonella* enterica subsp. enterica serovar Paratyphi B	3	.	*Enterobacteriales*
*Clostridium*	.	3	*Clostridiales*
*Peptoniphilus*	.	3	*Clostridiales*

1 only terminal clades with consistency scores < = −3 or > = 3 that were found using the EMBL or RDP 16S ribosomal databases as template are shown. Full consistency data are provided as Table S4 and Figure S4. Both annotations were included because well-annotated sequences by RDP are often not well annotated by EMBL and *vice versa* (Figure S4*C*).

## Discussion

The most striking observation from our current study was the dramatically different microbiomes in CRC tissue and adjacent non-malignant mucosa in 5 of the 6 investigated patients. To our surprise, however, we found no consistent overrepresentation of potential pathogenic bacteria in CRC tissue. In contrast, overrepresented species concerned members of the genera *Coriobacteridae*, *Roseburia*, *Fusobacterium* and *Faecalibacterium*, which are generally regarded as gut commensals with pro-biotic features. This suggests that the observed microbial shifts are caused by the quite dramatic physiological and metabolic alterations that result from colon carcinogenesis itself [38]–[40], and that these species may be regarded as CRC passengers. In fact, recent metabolomics studies revealed extremely altered nutritional conditions in the CRC tumor microenvironment compared to non-malignant mucosa [41]. The most prominent and consistent findings concerned a drastic decrease in glucose and pyruvate and an increase in lactate (low pH), amino acids, lipids and fatty acids. The intra-patient variability in microbiome alterations could possibly reflect the intra-individual variability in the CRC tumor microenvironment [42], resulting in the preferential recruitment of different classes of intestinal species. Notably, reduced numbers of *Collinsella* spp. and *Roseburia* spp. have previously been found in elderly subjects using non-steroidal anti-inflammatory drugs compared to non-users (NSAID [43]; suggesting that these bacteria need inflammatory niches to optimally colonize the bowel wall. Interestingly, the genera *Roseburia*, *Fusobacterium* and *Faecalibacterium*, which are moderately enriched in tumors belong to the major butyrate producing intestinal bacteria. Butyrate is thought to be protective against CRC by inducing a p21-dependent cell cycle arrest resulting in an increased apoptosis rate of carcinogenic cells [44]. The effects of butyrate are however still under debate as tumor inhibition may for instance be restricted to the early phases of carcinogenesis. Markedly, it has been shown that *Faecalibacterium prausnitzii* secretes anti-inflammatory factors that block NF-κB activation and IL-8 production in an experimental animal model for Crohn's disease [45]. Moreover, patients with inflammatory bowel disease, who are at increased risk for CRC, have been associated with lower numbers of *F. prausnitzii* in the intestinal population [46]. Finally, *Slackia* spp. are known to convert dietary isoflavones into more potent anti-oxidants [47] capable of inducing apoptotic pathways in tumor cells [48]. Thus in view of our current data, we could draw the conclusion that the CRC microenvironment is preferably colonized by intestinal bacteria with anti-tumorigenic and anti–carcinogenic properties, which thereby may prevent rapid progression of this disease. However, one could also argue that for instance butyrate provides an additional energy source for tumor cells, while dampening the inflammatory response stops the innate immune system from attacking the nascent tumor. Thus, both tumor suppressing or tumor promoting scenarios may be possible outcomes of the differential colonization of CRC tissue and further, detailed investigations, will be required to elucidate this issue.

Another remarkable observation concerned the decreased presence of members of the *Enterobacteriaceae*, such as *Citrobacter*, *Shigella*, *Cronobacter*, *Kluyvera*, *Serratia* and *Salmonella* spp. in tumour tissue of the investigated CRC patients. This data may suggest that these bacteria are part of the intrinsic microbiome of CRC patients, but outcompeted by the above mentioned commensal-like bacteria upon disease progression. Although we realize that in the absence of a large reference database of mucosal microbiomes from healthy individuals it is difficult to draw conclusions on this observation, we would like to take the opportunity to shortly review why Enterobacterial intestinal colonization could be associated with an increased risk for CRC. First, metagenomic inventories of the human intestinal microbiome showed that *Salmonella*, *Citrobacter*, and *Cronobacter* were among the low abundant intestinal species or were even completely absent in healthy individuals [15], [49], which is fully in-line with their pathogenic character [50]. Contrarily, this bacterial family was detectably present in non-malignant colonic mucosa samples from CRC patients [36], while Shen and colleagues recently showed that *Shigella* spp displayed an increased abundance in the intrinsic (non-malignant) microbiome of adenoma patients [51]. Importantly, the potential of Enterobacteria to initiate CRC has already been shown for *Citrobacter* species in an animal model [52], and it is thought that this increased susceptibility for CRC is caused by an asymptomatic, but chronic, inflammatory response in the colonic mucosa [53]. Additionally, several Enterobacterial strains produce DNA damaging genotoxins [54] and may thereby actively contribute to the accumulation of mutations that characterize the adenoma-carcinoma sequence [55]. In this context, our data may further suggest that upon CRC progression, the tumor microenvironment changes in such a way that Enterobacteria are replaced by commensal-like species or bacteria with proposed probiotic properties that have increased access to and/or can more efficiently forage in the altered tumor microenvironment. The disappearance of CRC-driving pathogenic bacteria from advanced CRC tumor tissue may be analogous to what has been reported for *Helicobacter pylori* during gastric cancer progression [56].

Altogether, our study provides an important first glimpse of the CRC-associated microbiome and indicates a highly dynamic relationship between intestinal bacteria and developing tumours. Nonetheless, many open questions need to be addressed in future studies, including deep microbiome analysis of extended groups of tumor samples from different disease stages, including adenomas and biopsies from a large set of non-cancer patients to serve as reference database. Furthermore, for diagnostic purposes it will be important to investigate how the local tumour-associated microbiome shifts relate to the fecal microbiota composition [57]. A more detailed analysis of CRC (meta-)genomes and bacterial transcriptomes is needed to pinpoint the genes that cause differential colonization in the tumor microenvironment and to better define high-risk microbial populations. Subsequently, high-risk and low-risk bacterial populations should be validated in (animal) models for sporadic CRC, and mechanisms of bacterial interference in CRC have to be unraveled in more detail. All-in-all, this sets the agenda for a new exciting era in colorectal cancer research, integrating microbiology and microbial ecology with tumor biology. This will lead us towards an increased understanding of the driving forces of CRC, as well as novel microbiome-related diagnostic tools and therapeutic interventions.

## Supporting Information

Figure S1 RISA fingerprinting of CRC tissue and non-malignant adjacent mucosa. The intergenic spacer region between the 16S and 23S rRNA genes was amplified with conserved primer pairs (Table S1) and analyzed using an Agilent Bioanalyser. Patient characteristics can be found in Table 1; off, non-malignant tissue; on, tumor tissue.
(PDF)

Figure S2 ***A***, Read lengths per sample, with X_off_ and X_on_ coming from off-tumor and on-tumor samples in subject X, the data was derived from the read lengths post-processing via the RDP pyropipeline. ***B***, Rarefraction curves for each sample, the sample key is the same as use for Figure S1. These curves were generated using MOTHUR, cut off values are shown. ***C***, Venn diagram for paired samples generated using MOTHUR, each diagram shows the OTUs (at 0.03% cut-off) shared and those unique to each sample from on and off tumor. ***D***, Principle component analysis of the annotated 16S rRNA sequence data generated for on and off tumor samples of each patient (A–F), was plotted as an 0.5×0.5 interval density distribution. The color coding shows the natural logarithm of the densities in each segment. Where there are significant differences (P<0.05) between tumor tissue and adjacent off-tumor mucosa, a white cross is shown in that segment. The taxonomic groups contributing to the most densely populated segments are shown (and the numbers of sequences contributing are shown in parentheses).
(PDF)

Figure S3 Taxonomic affiliation of the 16S rRNA gene reads for each paired sample set from subjects ***A***–***F*** and the combined samples (***G***). The figures were generated using MEGAN and show in which PHYLA (piecharts) and families (barcharts) the main alterations of levels of reads are found. In the barchart only families which were greater than 1% of the community are shown.
(PDF)

Figure S4 Consistent biases in microbiota between the on- and off-tumor samples. The overall consistency scores between +6 (green) and −6 (red) reflects how consistently clades were enriched across six patients. Trees were visualized with iTOL ***A***, Consistent clades derived from the EMBL annotation of SILVA sequences. ***B***, Consistent clades derived from the RDP annotation of SILVA sequences. ***C***, Differential annotation depth of the SSU rRNA sequences in the SILVA database by EMBL and RDP. Low annotation depth means little resolution: many sequences are either well annotated by RDP (bottom right) or by EMBL (top left).
(PDF)

Table S1 Primers used in this study.
(PDF)

Table S2 MOTHUR diversity indices of bacterial communities in samples on- and off-tumor (X_on_ and X_off_, respectively).
(PDF)

Table S3 Genus level comparison generated with the RDP library compare tool. Values indicate the number of 16S rRNA pyrosequencing reads that map to the listed genus. Only significant differences are shown (P<0.05). A–F: patients; X_off_: off-tumor tissue; X_on_: on-tumor tissue.
(PDF)

Table S4 Consistency scores for CRC associated microbiome shifts.
(XLS)
