# Supplementary material for: Towards the Human Colorectal Cancer Microbiome
Source: PLoS One. 2011 May 24;6(5):e20447. doi: 10.1371/journal.pone.0020447 (PMC3101260; doi:10.1371/journal.pone.0020447)

Figure S1. RISA fingerprinting of CRC tissue and non-malignant adjacent mucosa. The intergenic spacer region between the 16S and 23S rRNA genes was amplified with conserved primer pairs (Table S1) and analyzed using an Agilent Bioanalyser. Patient characteristics can be found in Table 1; off, non-malignant tissue; on, tumor tissue.

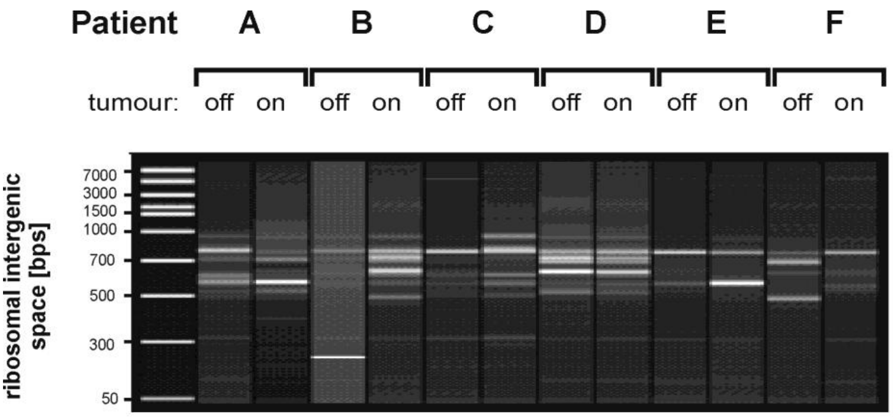

Supplement: Figure S1 — RISA fingerprinting of CRC tissue and non-malignant adjacent mucosa. The intergenic spacer region between the 16S and 23S rRNA genes was amplified with conserved primer pairs (Table S1) and analyzed using an Agilent Bioanalyser. Patient characteristics can be found in Table 1; off, non-malignant tissue; on, tumor tissue. (PDF) [file pone.0020447.s001.pdf]
