# Supplementary material for: Towards the Human Colorectal Cancer Microbiome
Source: PLoS One. 2011 May 24;6(5):e20447. doi: 10.1371/journal.pone.0020447 (PMC3101260; doi:10.1371/journal.pone.0020447)
Supplement: Figure S2 — A, Read lengths per sample, with Xoff and Xon coming from off-tumor and on-tumor samples in subject X, the data was derived from the read lengths post-processing via the RDP pyropipeline. B, Rarefraction curves for each sample, the sample key is the same as use for Figure S1. These curves were generated using MOTHUR, cut off values are shown. C, Venn diagram for paired samples generated using MOTHUR, each diagram shows the OTUs (at 0.03% cut-off) shared and those unique to each sample from on and off tumor. D, Principle component analysis of the annotated 16S rRNA sequence data generated for on and off tumor samples of each patient (A–F), was plotted as an 0.5×0.5 interval density distribution. The color coding shows the natural logarithm of the densities in each segment. Where there are significant differences (P<0.05) between tumor tissue and adjacent off-tumor mucosa, a white cross is shown in that segment. The taxonomic groups contributing to the most densely populated segments are shown (and the numbers of sequences contributing are shown in parentheses). (PDF) [file pone.0020447.s002.pdf]

Figure S2. ***A**,* Read lengths per sample, with X_{off} and X_{on} coming from off-tumor and on-tumor samples in subject X, the data was derived from the read lengths post-processing via the RDP pyropipeline [1]. ***B**,* Rarefraction curves for each sample, the sample key is the same as use for Figure S1. These curves were generated using MOTHUR [2,3], cut off values are shown. ***C***, Venn diagram for paired samples generated using MOTHUR, each diagram shows the OTUs (at 0.03% cut-off) shared and those unique to each sample from on and off tumor. ***D***, Principle component analysis of the annotated 16S rRNA sequence data generated for on and off tumor samples of each patient (A-F), was plotted as an 0.5 x 0.5 interval density distribution. The color coding shows the natural logarithm of the densities in each segment. Where there are significant differences (P <0.05) between tumor tissue and adjacent off-tumor mucosa, a white cross is shown in that segment. The taxonomic groups contributing to the most densely populated segments are shown (and the numbers of sequences contributing are shown in parentheses).

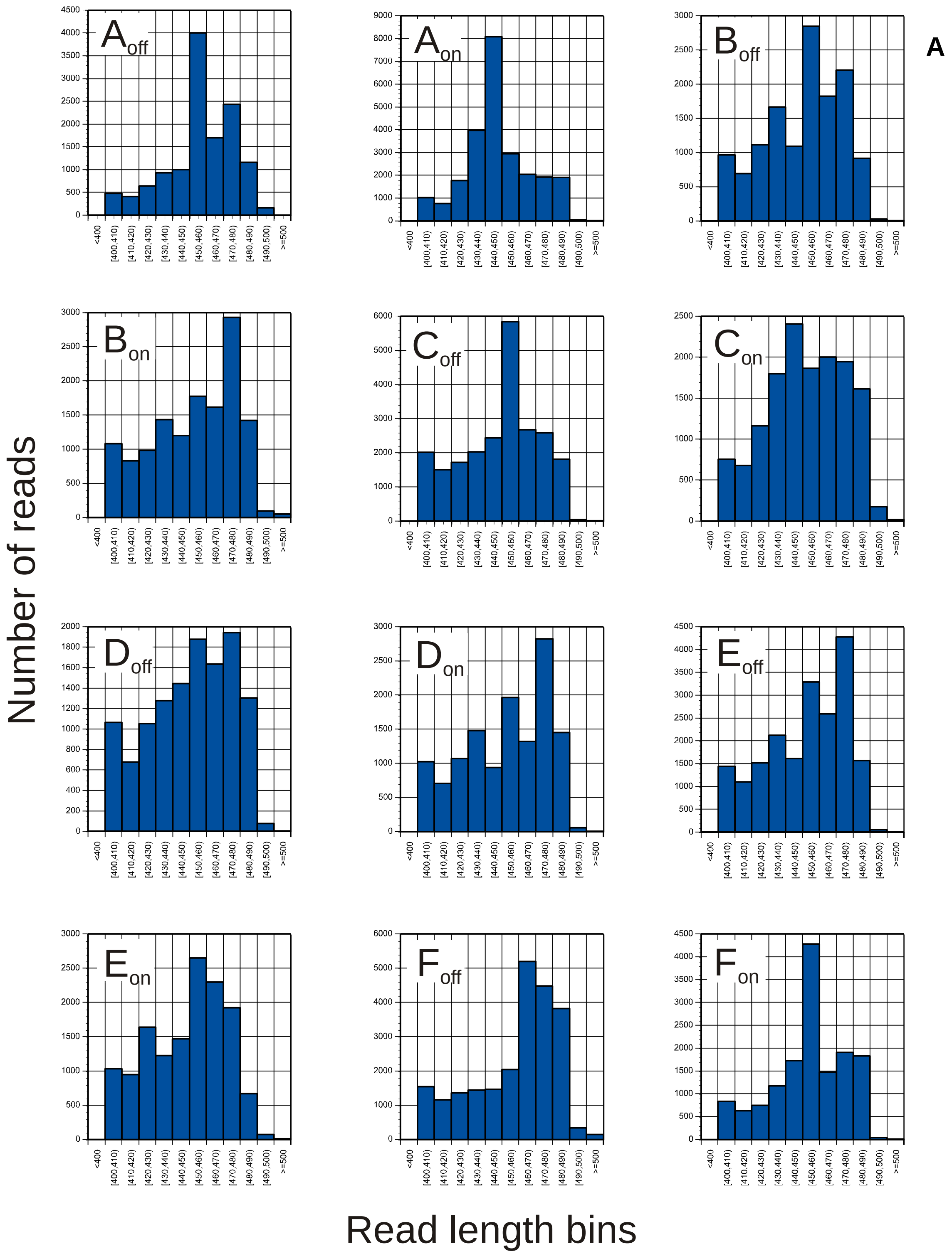

B

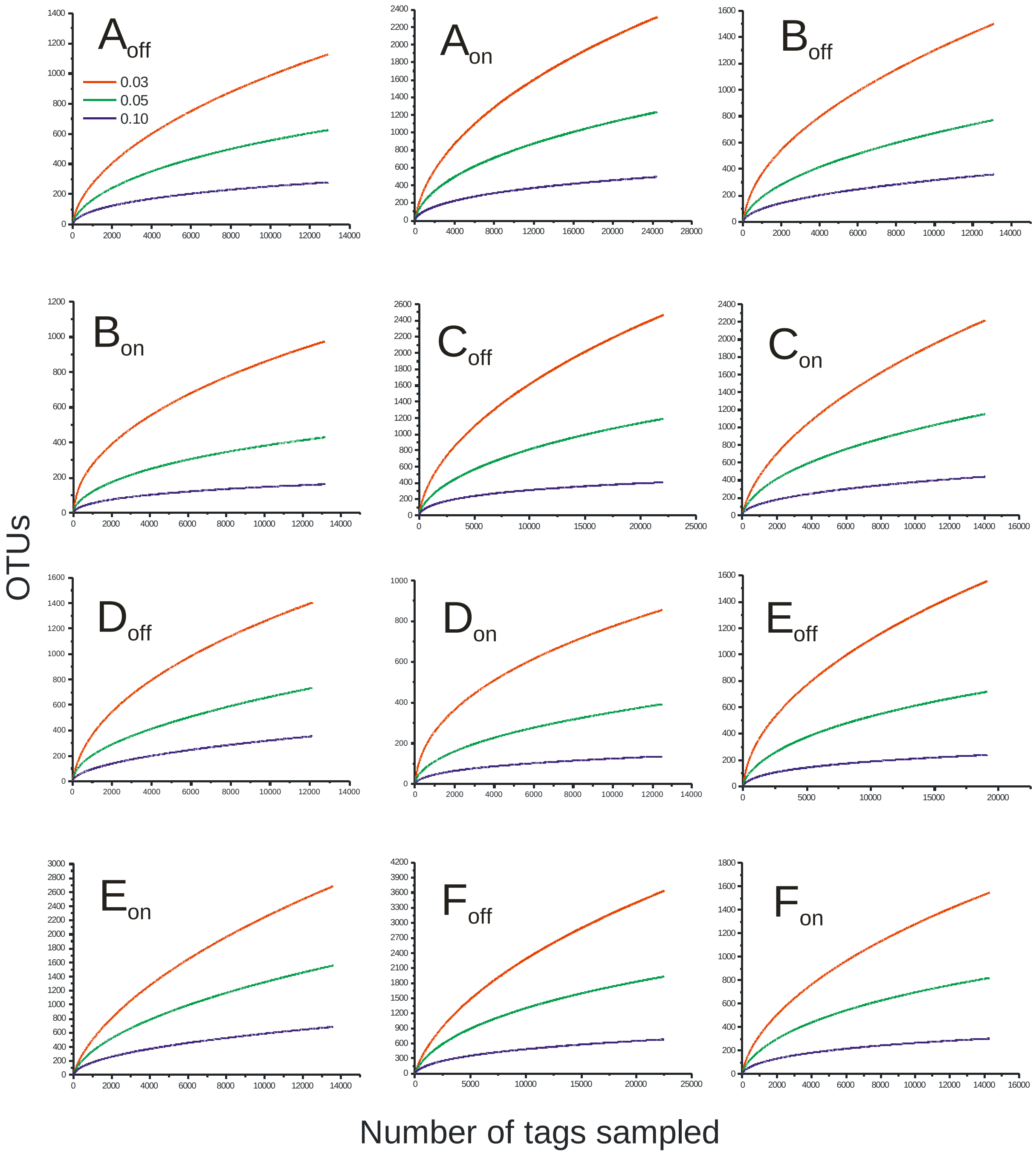

A off
0.03
0.05
0.10
A on
B off
B on
C off
C on
D off
D on
E off
E on
F off
F on
OTUs
Number of tags sampled

C

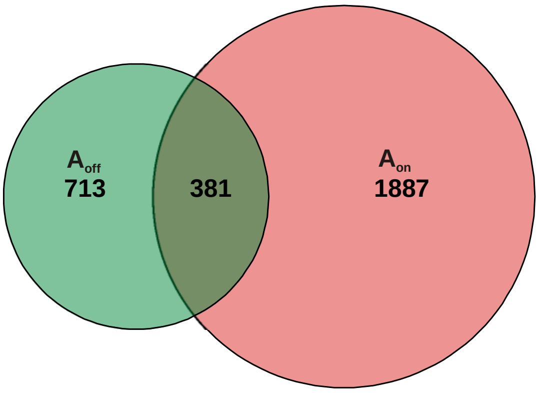

The number of species in group A_{off} is 1094
The number of species in group A_{on} is 2268
The number of species shared between groups A_{off} and A_{on} is 381
% of species that are shared in groups A_{off} and A_{on} is 12%
The total richness for all groups is 2981

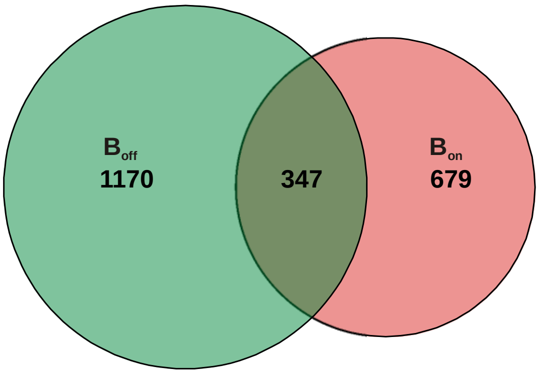

The number of species in group B_{off} is 1517
The number of species in group B_{on} is 1026
The number of species shared between groups B_{off} and B_{on} is 347
% of species that are shared in groups B_{off} and B_{on} is 16%
The total richness for all groups is 2196

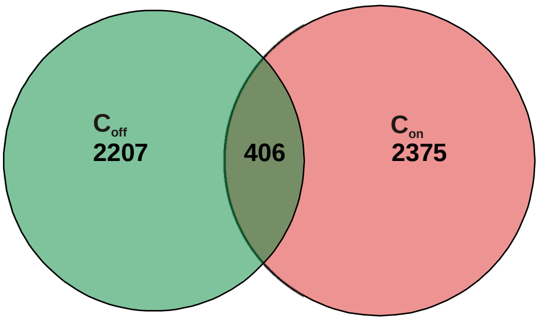

The number of species in group C_{off} is 2613
The number of species in group C_{on} is 2781
The number of species shared between groups C_{off} and C_{on} is 406
% of species that are shared in groups C_{off} and C_{on} is 8%
The total richness for all groups is 4988

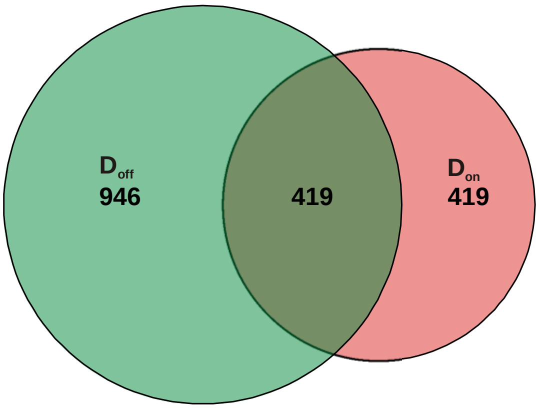

The number of species in group D_{off} is 1439
The number of species in group D_{on} is 912
The number of species shared between groups D_{off} and D_{on} is 493
% of species that are shared in groups D_{off} and D_{on} is 27%
The total richness for all groups is 1858

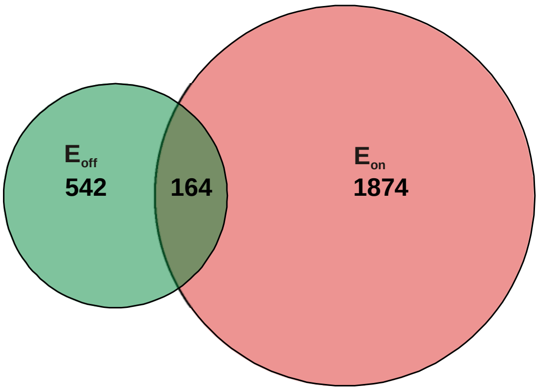

The number of species in group E_{off} is 706
The number of species in group E_{on} is 2038
The number of species shared between groups E_{off} and E_{on} is 164
% of species that are shared in groups E_{off} and E_{on} is 6%
The total richness for all groups is 2580

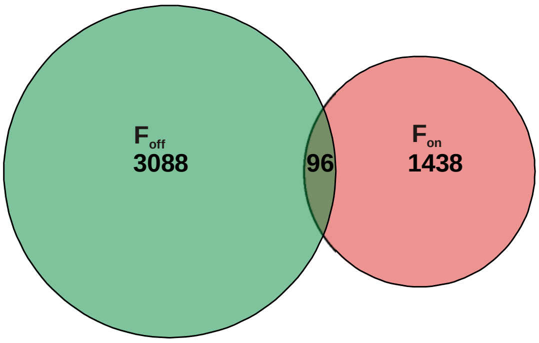

The number of species in group F_{off} is 3184
The number of species in group F_{on} is 1534
The number of species shared between groups F_{off} and F_{on} is 96
% of species that are shared in groups F_{off} and F_{on} is 2%
The total richness for all groups is 4622

D

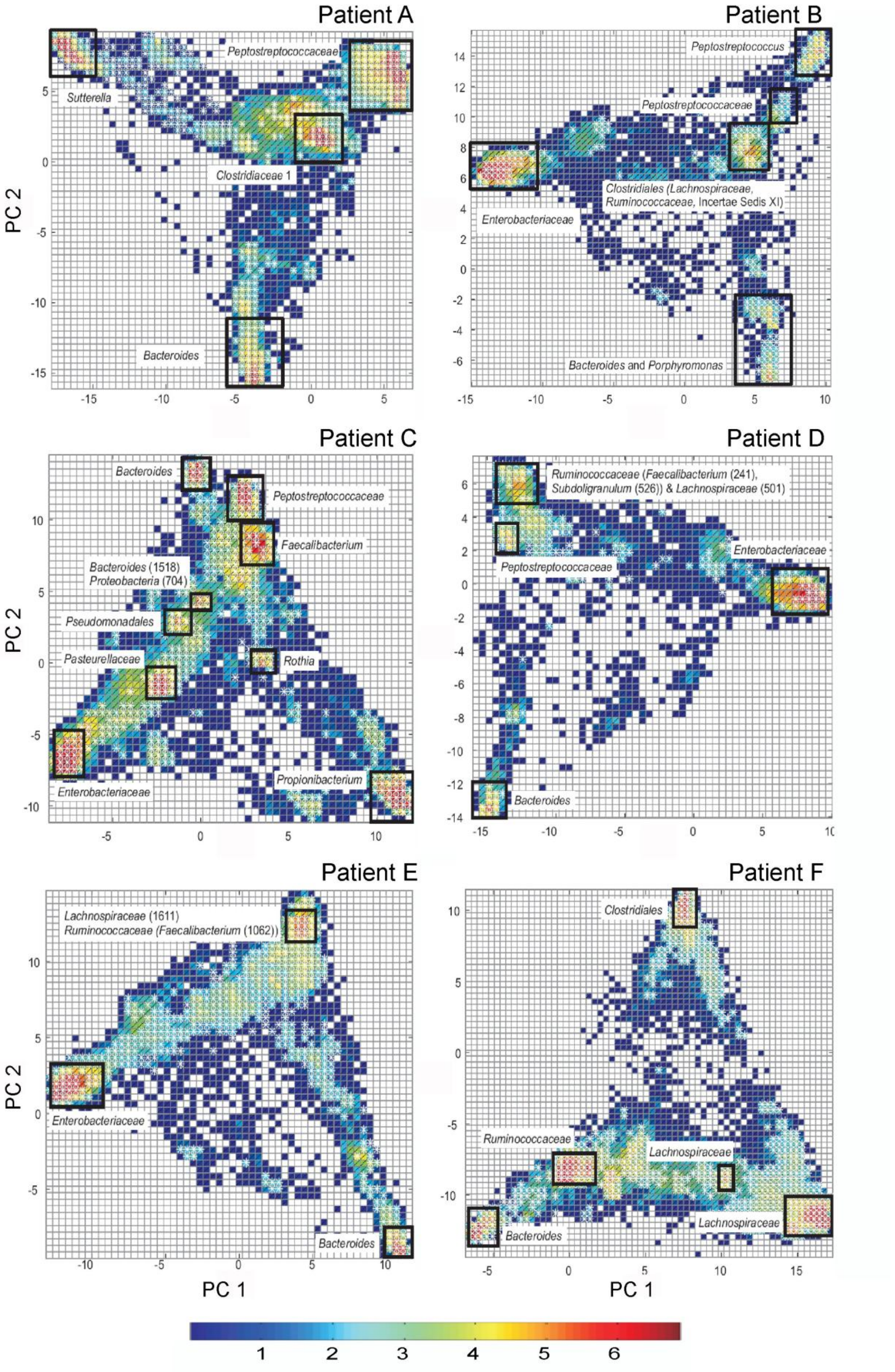
Patient A
Peptostreptococcaceae
Sutterella
Clostridiaceae 1
Bacteroides
Patient B
Peptostreptococcus
Peptostreptococcaceae
Clostridiales (Lachnospiraceae, Ruminococcaceae, Incertae Sedis XI)
Enterobacteriaceae
Bacteroides and Porphyromonas
Patient C
Bacteroides
Peptostreptococcaceae
Faecalibacterium
Bacteroides (1518)
Proteobacteria (704)
Pseudomonadales
Pasteurellaceae
Rothia
Enterobacteriaceae
Propionibacterium
Patient D
Ruminococcaceae (Faecalibacterium (241), Subdoligranulum (526)) & Lachnospiraceae (501)
Peptostreptococcaceae
Enterobacteriaceae
Bacteroides
Patient E
Lachnospiraceae (1611)
Ruminococcaceae (Faecalibacterium (1062))
Enterobacteriaceae
Bacteroides
Patient F
Clostridiales
Ruminococcaceae
Lachnospiraceae
Lachnospiraceae
Bacteroides
PC 1
PC 2
1
2
3
4
5
6

REFERENCES

1 Cole JR et al. The Ribosomal Database Project: improved alignments and new tools for rRNA analysis. Nucl Acids Res 2009;37:D141-145.
2. Schloss PD et al. Introducing mothur: Open Source, Platform-independent, Community-supported Software for Describing and Comparing Microbial Communities. Appl Environ Microbiol 2009: AEM.01541-09.
3. Huson DH et al. Methods for comparative metagenomics. BMC Bioinformatics 2009;10 Suppl 1:S12.
