# Supplementary material for: Towards the Human Colorectal Cancer Microbiome
Source: PLoS One. 2011 May 24;6(5):e20447. doi: 10.1371/journal.pone.0020447 (PMC3101260; doi:10.1371/journal.pone.0020447)
Supplement: Figure S3 — Taxonomic affiliation of the 16S rRNA gene reads for each paired sample set from subjects A–F and the combined samples (G). The figures were generated using MEGAN and show in which PHYLA (piecharts) and families (barcharts) the main alterations of levels of reads are found. In the barchart only families which were greater than 1% of the community are shown. (PDF) [file pone.0020447.s003.pdf]

Figure S3. Taxonomic affiliation of the 16S rRNA gene reads for each paired sample set from subjects ***A*-*F*** and the combined samples (***G***). The figures were generated using MEGAN [1,2] and show in which PHYLA (piecharts) and families (barcharts) the main alterations of levels of reads are found. In the barchart only families which were greater than 1% of the community are shown.

Subject A

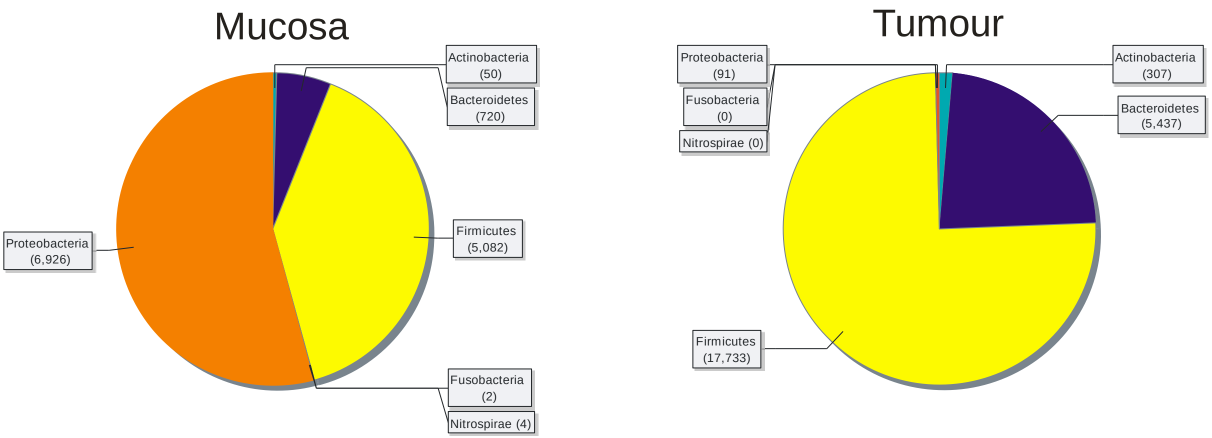

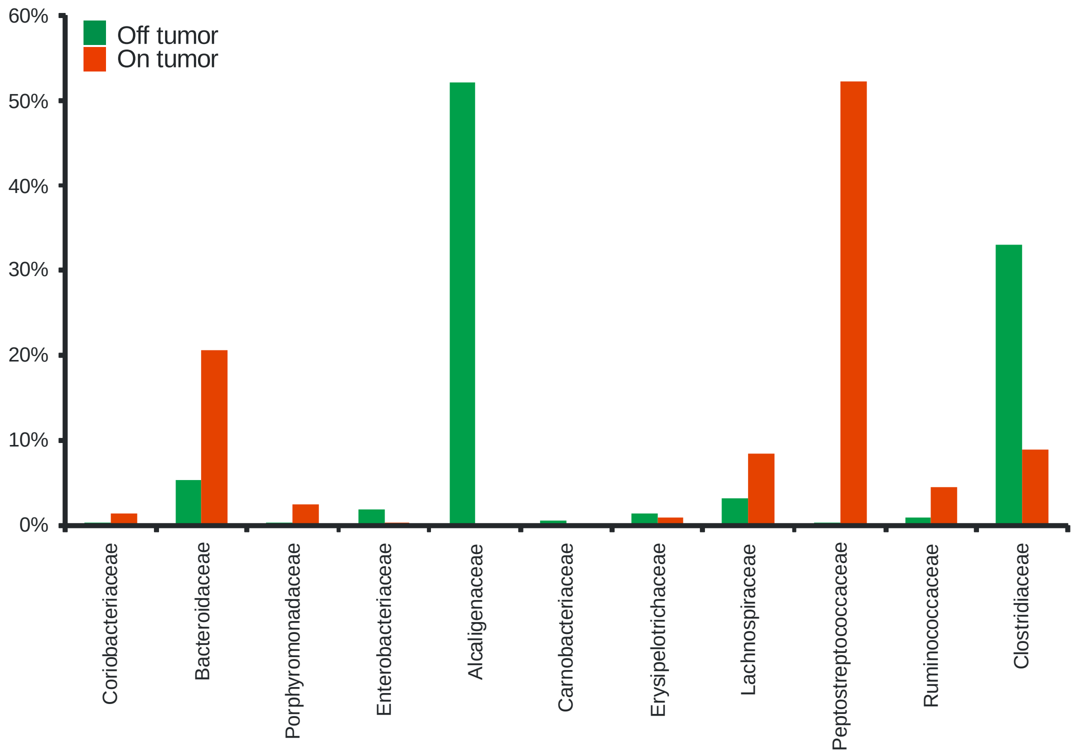

Subject B

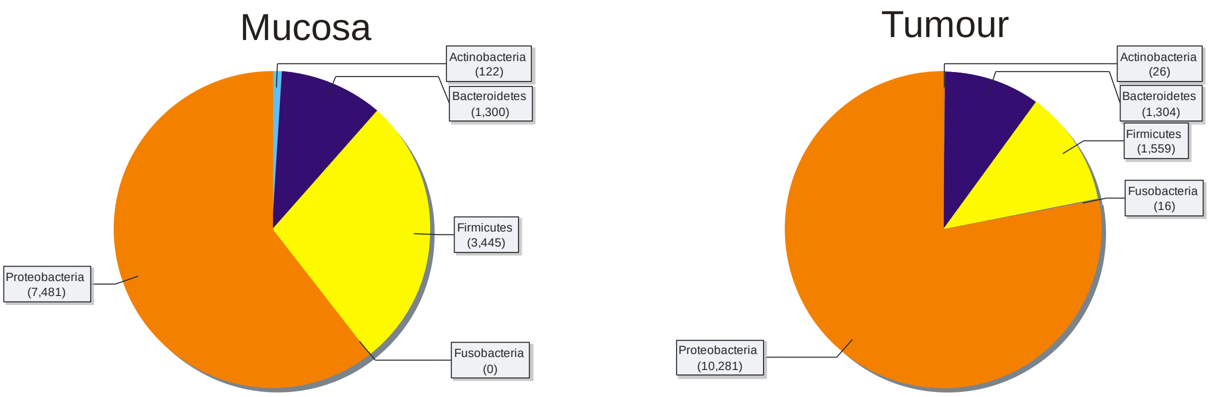
Mucosa
Actinobacteria (122)
Bacteroidetes (1,300)
Firmicutes (3,445)
Fusobacteria (0)
Proteobacteria (7,481)
Tumour
Actinobacteria (26)
Bacteroidetes (1,304)
Firmicutes (1,559)
Fusobacteria (16)
Proteobacteria (10,281)

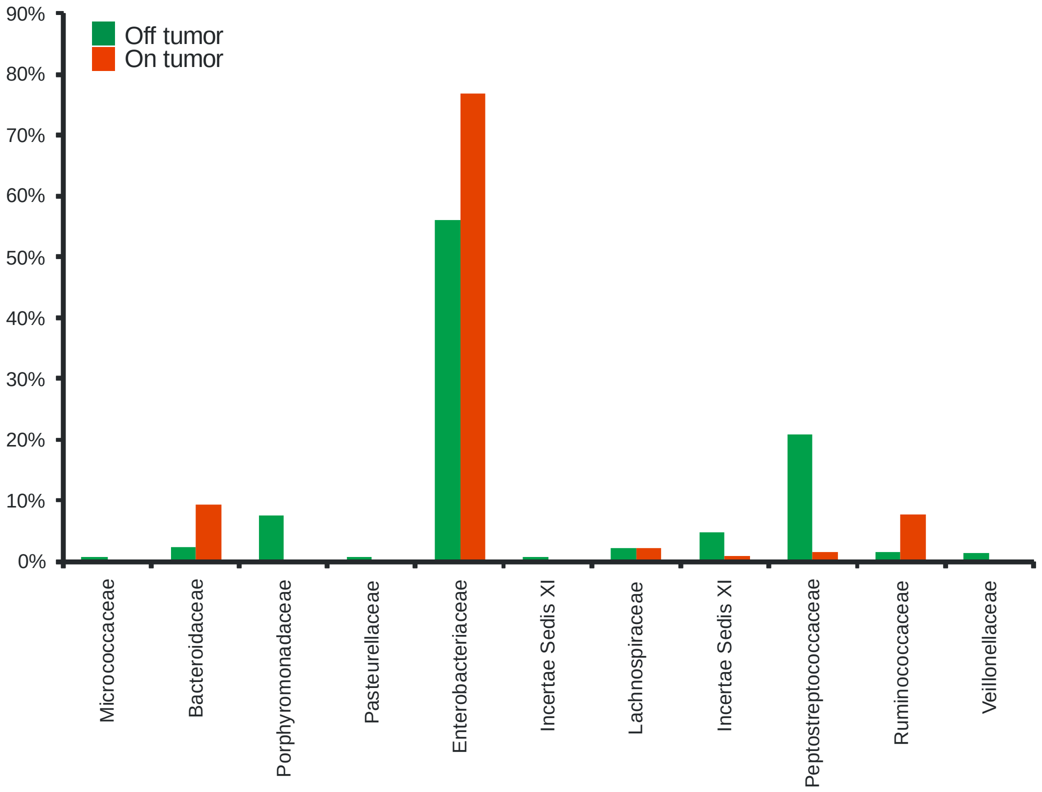
90%
80%
70%
60%
50%
40%
30%
20%
10%
0%
Off tumor
On tumor
Micrococcaceae
Bacteroidaceae
Porphyromonadaceae
Pasteurellaceae
Enterobacteriaceae
Incertae Sedis XI
Lachnospiraceae
Incertae Sedis XI
Peptostreptococcaceae
Ruminococcaceae
Veillonellaceae

Subject C

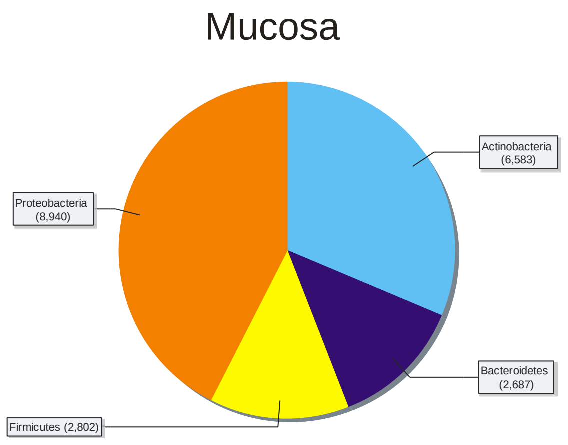
Mucosa
Actinobacteria (6,583)
Proteobacteria (8,940)
Bacteroidetes (2,687)
Firmicutes (2,802)

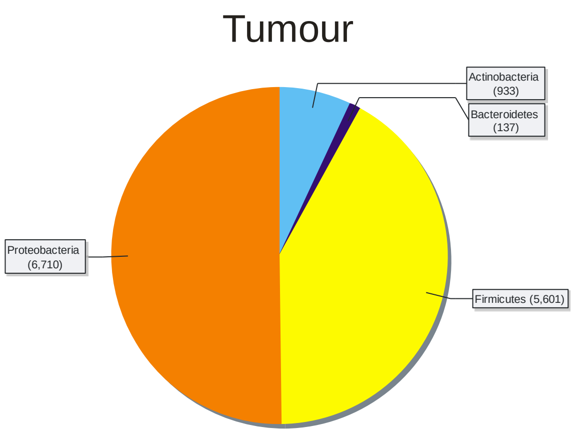
Tumour
Actinobacteria (933)
Bacteroidetes (137)
Proteobacteria (6,710)
Firmicutes (5,601)

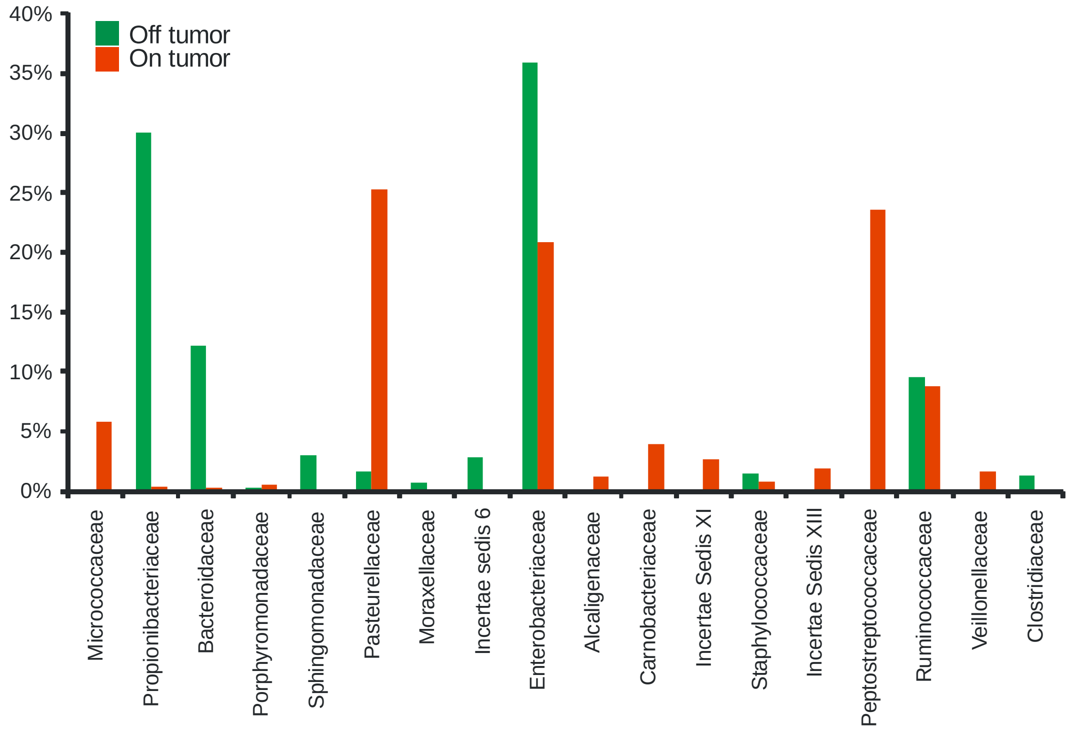
40%
35%
30%
25%
20%
15%
10%
5%
0%
Off tumor
On tumor
Micrococcaceae
Propionibacteriaceae
Bacteroidaceae
Porphyromonadaceae
Sphingomonadaceae
Pasteurellaceae
Moraxellaceae
Incertae sedis 6
Enterobacteriaceae
Alcaligenaceae
Carnobacteriaceae
Incertae Sedis XI
Staphylococcaceae
Incertae Sedis XIII
Peptostreptococcaceae
Ruminococcaceae
Veillonellaceae
Clostridiaceae

Subject D

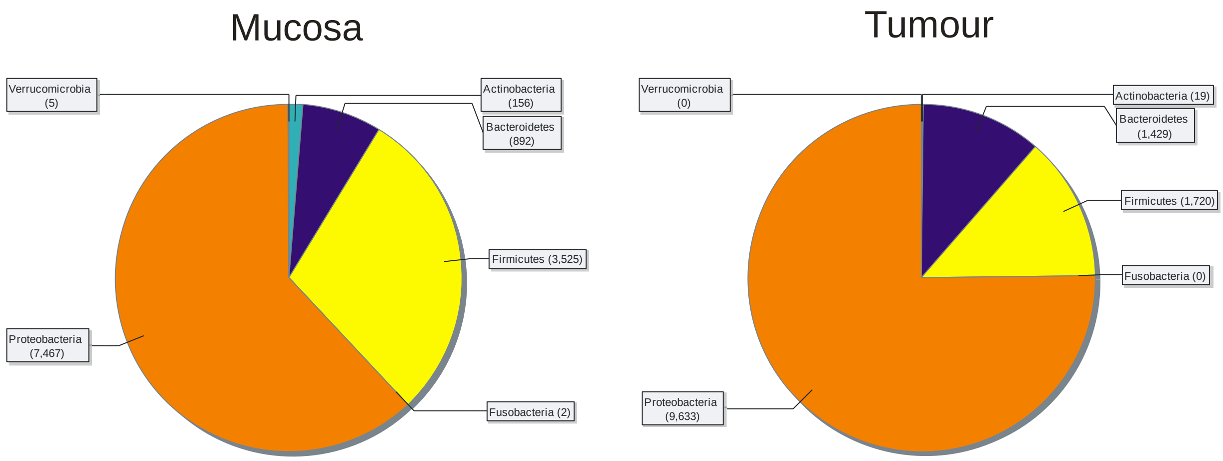
Mucosa
Verrucomicrobia (5)
Actinobacteria (156)
Bacteroidetes (892)
Firmicutes (3,525)
Proteobacteria (7,467)
Fusobacteria (2)
Tumour
Verrucomicrobia (0)
Actinobacteria (19)
Bacteroidetes (1,429)
Firmicutes (1,720)
Fusobacteria (0)
Proteobacteria (9,633)

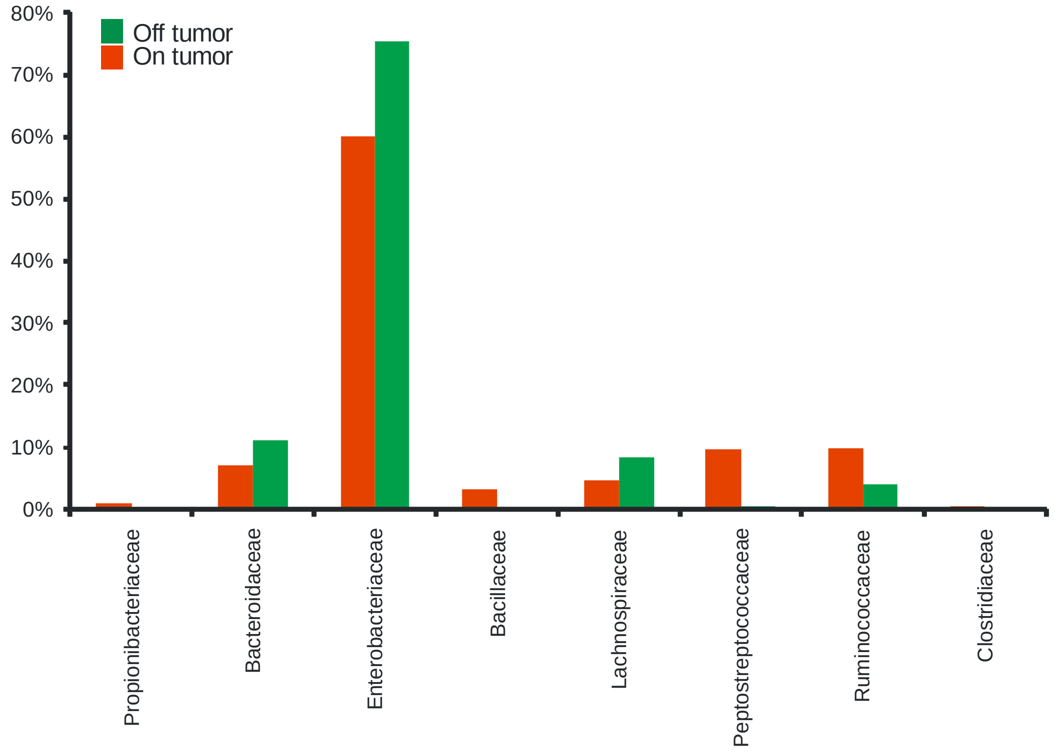
80%
70%
60%
50%
40%
30%
20%
10%
0%
Off tumor
On tumor
Propionibacteriaceae
Bacteroidaceae
Enterobacteriaceae
Bacillaceae
Lachnospiraceae
Peptostreptococcaceae
Ruminococcaceae
Clostridiaceae

Subject E

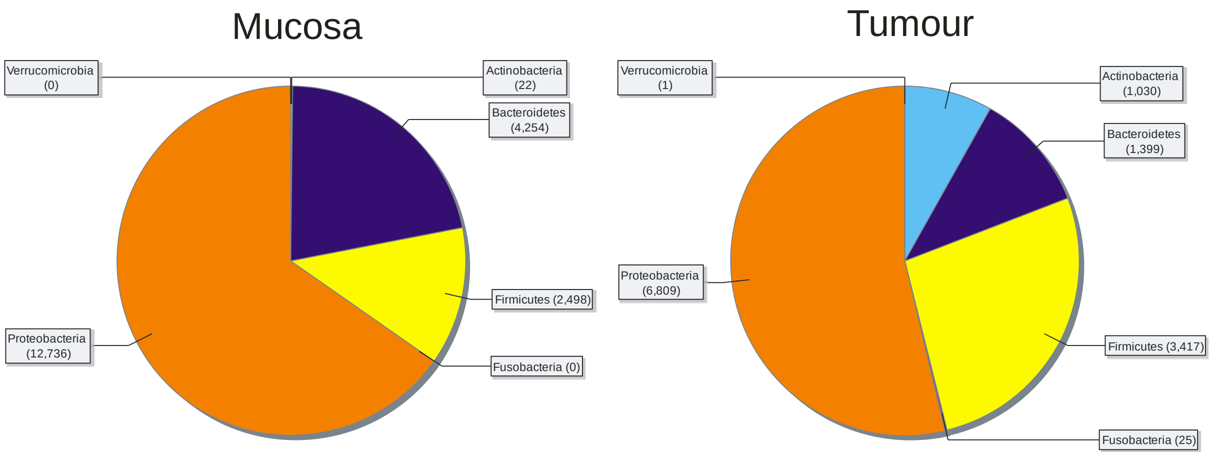
Mucosa
Verrucomicrobia (0)
Actinobacteria (22)
Bacteroidetes (4,254)
Firmicutes (2,498)
Fusobacteria (0)
Proteobacteria (12,736)
Tumour
Verrucomicrobia (1)
Actinobacteria (1,030)
Bacteroidetes (1,399)
Proteobacteria (6,809)
Firmicutes (3,417)
Fusobacteria (25)

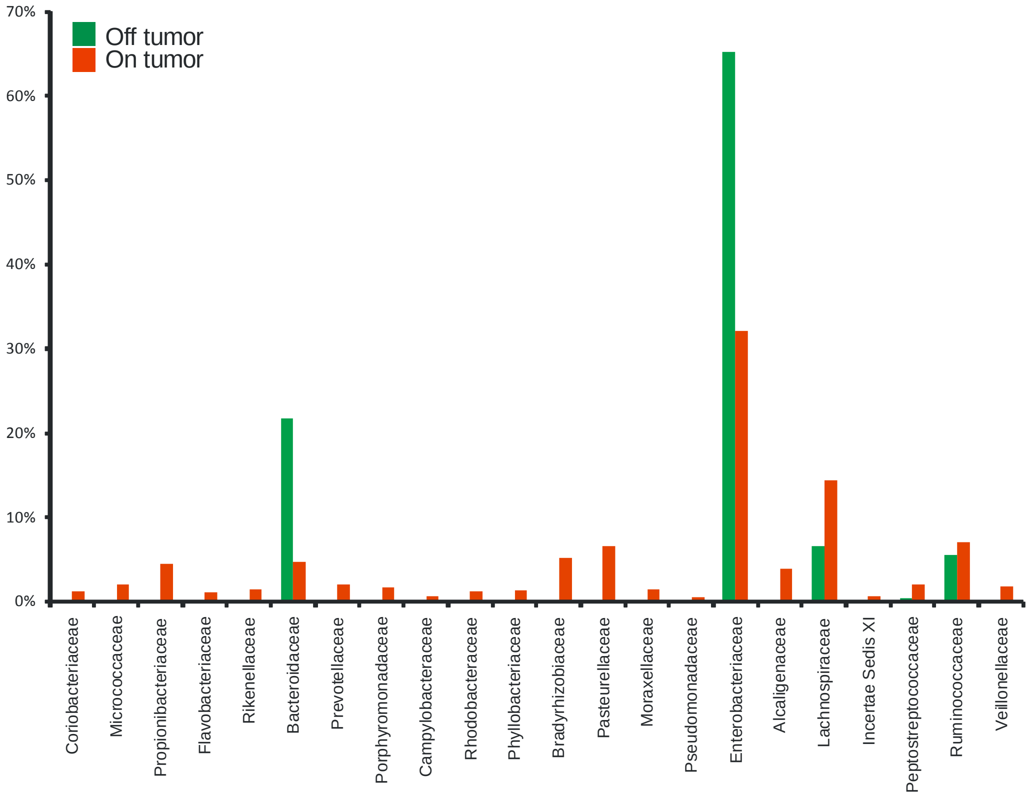
70%
60%
50%
40%
30%
20%
10%
0%
Off tumor
On tumor
Coriobacteriaceae
Micrococcaceae
Propionibacteriaceae
Flavobacteriaceae
Rikenellaceae
Bacteroidaceae
Prevotellaceae
Porphyromonadaceae
Campylobacteraceae
Rhodobacteraceae
Phyllobacteriaceae
Bradyrhizobiaceae
Pasteurellaceae
Moraxellaceae
Pseudomonadaceae
Enterobacteriaceae
Alcaligenaceae
Lachnospiraceae
Incertae Sedis XI
Peptostreptococcaceae
Ruminococcaceae
Veillonellaceae

Subject F

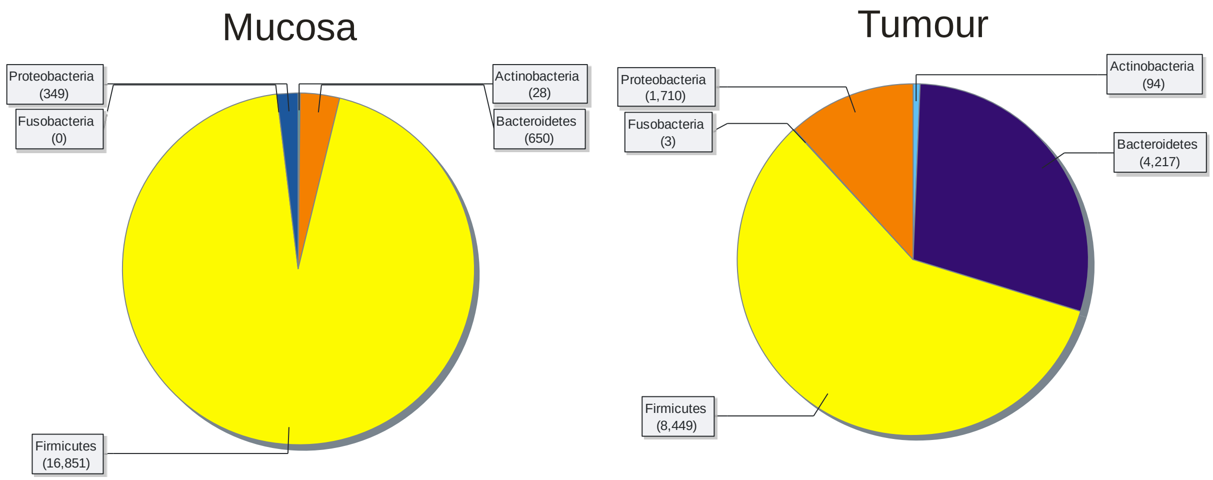
Mucosa
Proteobacteria (349)
Fusobacteria (0)
Actinobacteria (28)
Bacteroidetes (650)
Firmicutes (16,851)
Tumour
Proteobacteria (1,710)
Fusobacteria (3)
Actinobacteria (94)
Bacteroidetes (4,217)
Firmicutes (8,449)

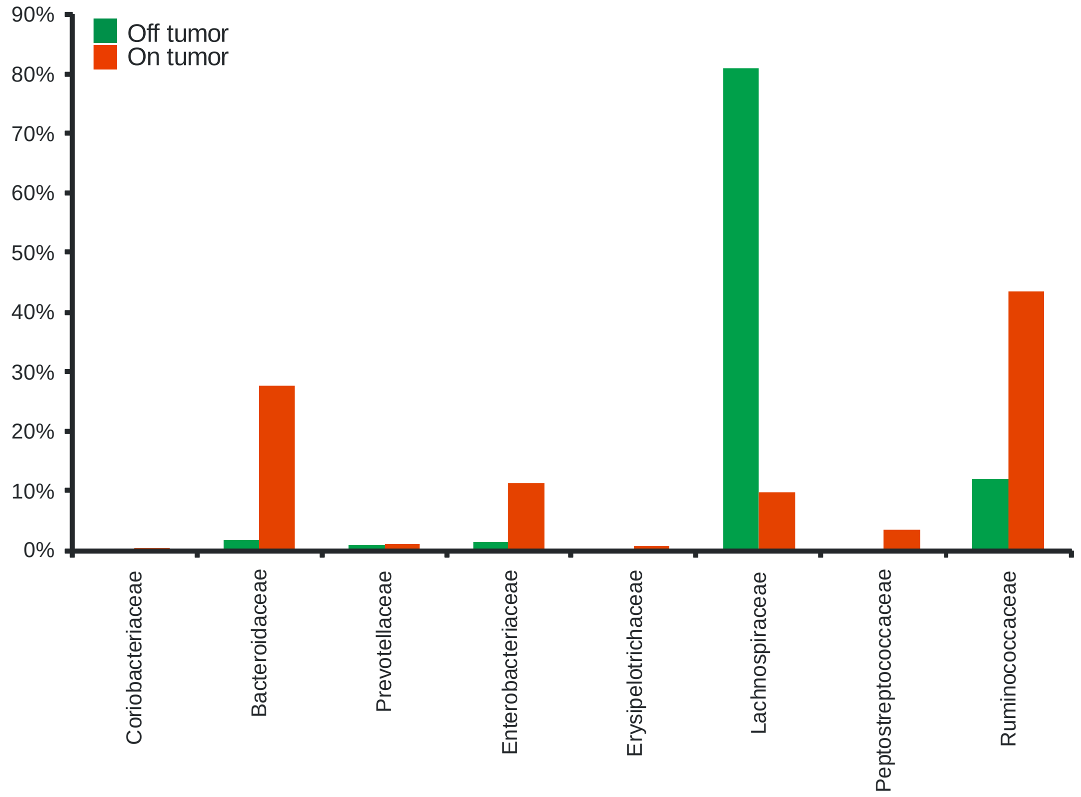
90%
80%
70%
60%
50%
40%
30%
20%
10%
0%
Off tumor
On tumor
Coriobacteriaceae
Bacteroidaceae
Prevotellaceae
Enterobacteriaceae
Erysipelotrichaceae
Lachnospiraceae
Peptostreptococcaceae
Ruminococcaceae

Combined

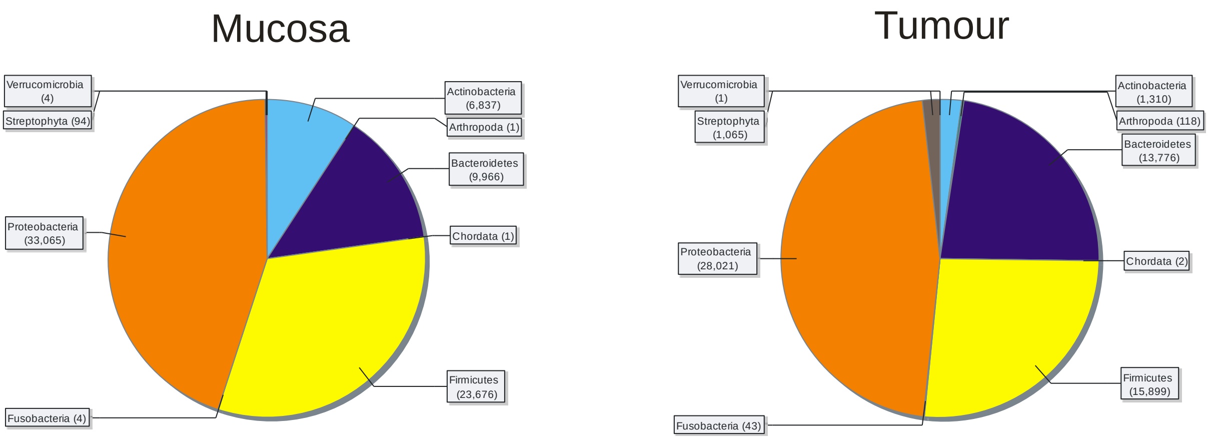

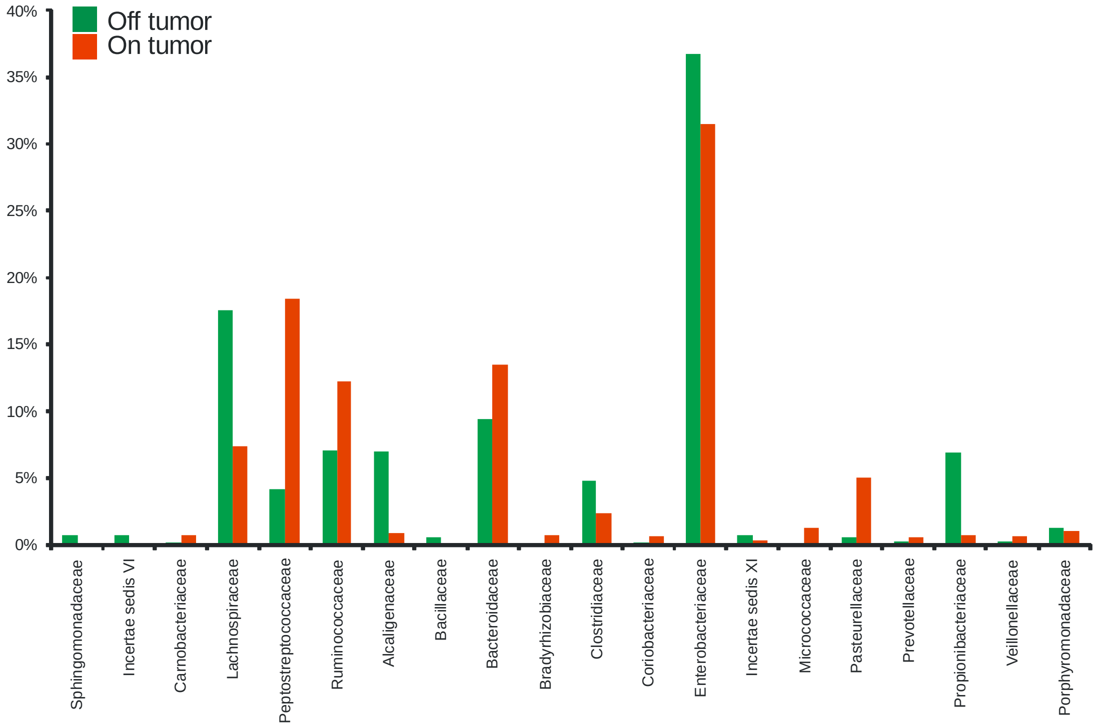

REFERENCES

1.. Huson DH et al. MEGAN analysis of metagenomic data. Genome Research 2007;17:377-386.
2. Letunic I et al. Interactive Tree Of Life (iTOL): an online tool for phylogenetic tree display and annotation. Bioinformatics 2007;23:127-128.
