# Supplementary material for: Towards the Human Colorectal Cancer Microbiome
Source: PLoS One. 2011 May 24;6(5):e20447. doi: 10.1371/journal.pone.0020447 (PMC3101260; doi:10.1371/journal.pone.0020447)
Supplement: Figure S4 — Consistent biases in microbiota between the on- and off-tumor samples. The overall consistency scores between +6 (green) and −6 (red) reflects how consistently clades were enriched across six patients. Trees were visualized with iTOL A, Consistent clades derived from the EMBL annotation of SILVA sequences. B, Consistent clades derived from the RDP annotation of SILVA sequences. C, Differential annotation depth of the SSU rRNA sequences in the SILVA database by EMBL and RDP. Low annotation depth means little resolution: many sequences are either well annotated by RDP (bottom right) or by EMBL (top left). (PDF) [file pone.0020447.s004.pdf]

Figure S4. Consistent biases in microbiota between the on- and off-tumor samples. The overall consistency scores between +6 (green) and -6 (red) reflects how consistently clades were enriched across six patients. Trees were visualized with iTOL [5] ***A***, Consistent clades derived from the EMBL annotation of SILVA sequences. ***B***, Consistent clades derived from the RDP annotation of SILVA sequences. ***C***, Differential annotation depth of the SSU rRNA sequences in the SILVA database [1] by EMBL and RDP. Low annotation depth means little resolution: many sequences are either well annotated by RDP (bottom right) or by EMBL (top left).

A

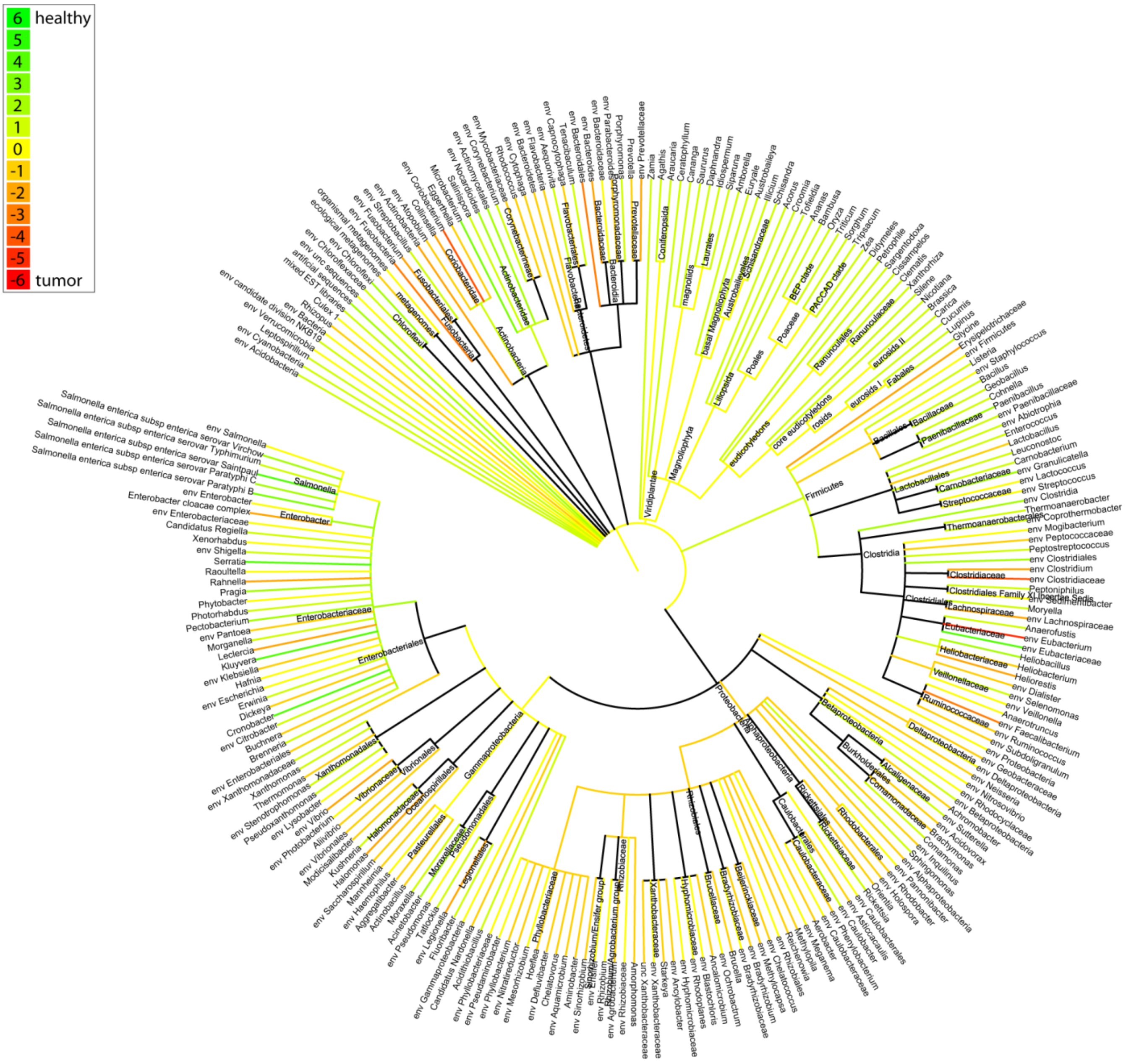

B

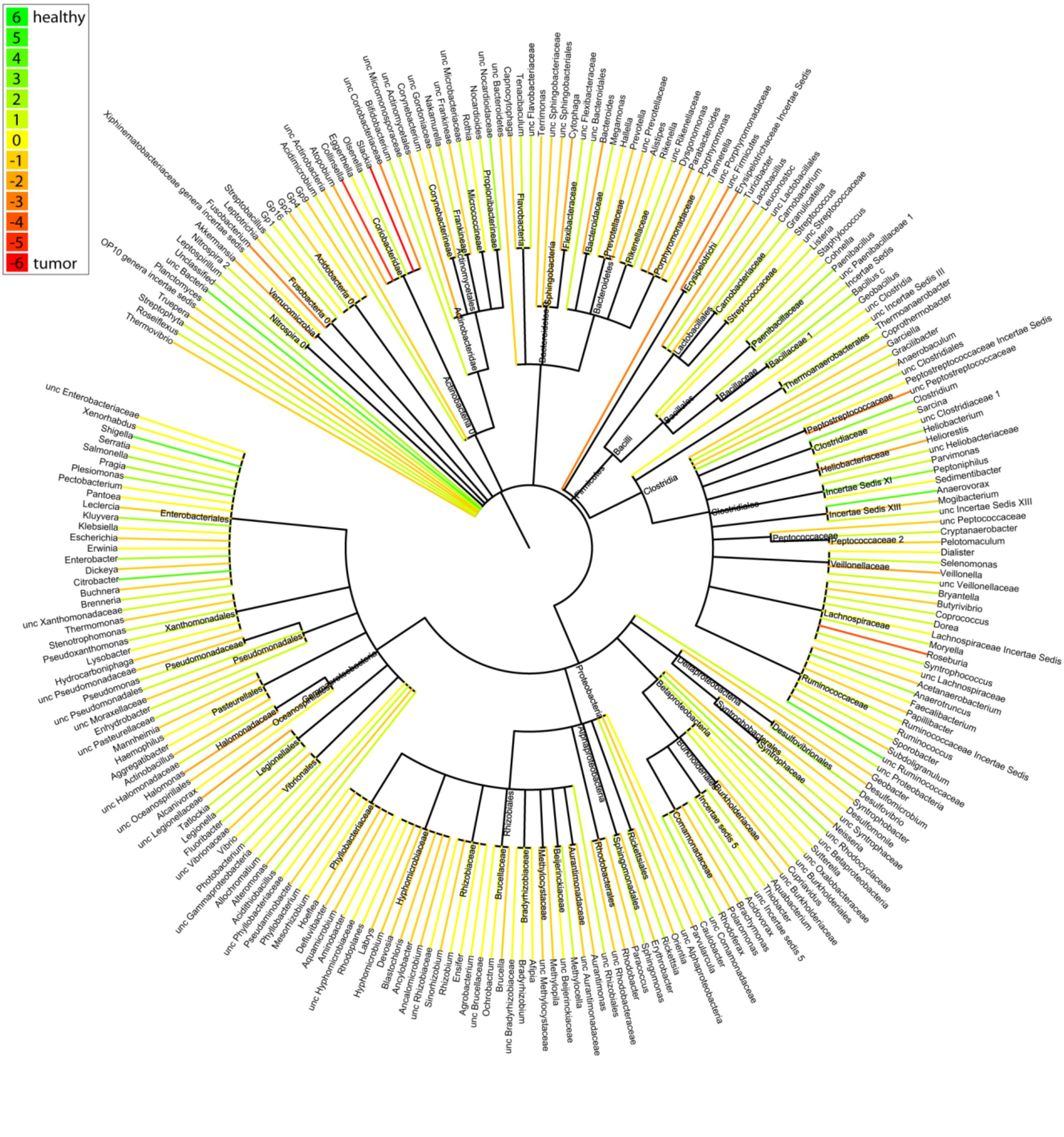
healthy
6
5
4
3
2
1
0
-1
-2
-3
-4
-5
-6
tumor
Xiphinematobacteriaceae genera incertae sedis
OP10 genera incertae sedis
Thermovibrio
Roseiflexus
Streptophyta
Truepera
Planctomyces
unc Bacteria
Unclassified
Leptospira
Nitrospira 2
Akkermansia
Fusobacterium
Leptotrichia
Streptobacillus
Gp1
Gp16
Gp6
Gp4
Gp2
Gp9
Acidimicrobium
unc Actinobacteria
Atopobium
Collinsella
Eggerthella
Olsenella
Slackia
unc Coriobacteriaceae
Bifidobacterium
unc Micromonosporaceae
unc Actinomycetales
Corynebacterium
unc Gordoniaceae
Nakamurella
unc Frankineae
unc Microbacteriaceae
Rothia
Nocardioides
unc Nocardioidaceae
unc Bacteroidetes
Capnocytophaga
Tenacibaculum
unc Flavobacteriaceae
Terrimonas
unc Sphingobacteriaceae
unc Sphingobacteriales
Cytophaga
unc Flexibacteraceae
Bacteroides
unc Bacteroidales
Megamonas
Hallella
Prevotella
unc Prevotellaceae
Alistipes
Rikenella
unc Rikenellaceae
Dysgonomonas
Parabacteroides
Porphyromonas
Tannerella
unc Porphyromonadaceae
unc Firmicutes
Erysipelotrichaceae Incertae Sedis
Turicibacter
Lactobacillus
Leuconostoc
unc Lactobacillales
Carnobacterium
Granulicatella
Streptococcus
unc Streptococcaceae
Listeria
Staphylococcus
Cohnella
Paenibacillus
unc Paenibacillaceae 1
Incertae Sedis
Bacillus c
Geobacillus
unc Clostridia
unc Incertae Sedis III
Thermoanaerobacter
Coprothermobacter
Garciella
Gracilibacter
Anaerobaculum
unc Clostridiales
Peptostreptococcaceae Incertae Sedis
unc Peptostreptococcaceae
Clostridium
Sarcina
unc Clostridiaceae 1
Heliobacterium
Heliorestis
unc Heliobacteriaceae
Parvimonas
Peptoniphilus
Sedimentibacter
Anaerovorax
Mogibacterium
unc Incertae Sedis XIII
unc Peptococcaceae
Cryptanaerobacter
Pelotomaculum
Dialister
Selenomonas
Veillonella
unc Veillonellaceae
Bryantella
Butyrivibrio
Coprococcus
Dorea
Lachnospiraceae Incertae Sedis
Moryella
Roseburia
Syntrophococcus
unc Lachnospiraceae
Acetanaerobacterium
Anaerotruncus
Faecalibacterium
Papillibacter
Ruminococcus
Ruminococcaceae Incertae Sedis
Sporobacter
Subdoligranulum
unc Ruminococcaceae
unc Proteobacteria
Geobacter
Desulfomicrobium
Desulfovibrio
Syntrophobacter
Desulfomonile
unc Syntrophaceae
Neisseria
unc Rhodocyclaceae
unc Betaproteobacteria
unc Oxalobacteraceae
Sutterella
unc Burkholderiales
Cupriavidus
unc Burkholderiaceae
Aquabacterium
unc Incertae sedis 5
Thiobacter
Acidovorax
Brachymonas
Polaromonas
Rhodoferax
unc Comamonadaceae
Caulobacter
Parvularcula
unc Alphaproteobacteria
Orientia
Rickettsia
Erythrobacter
Sphingomonas
Paracoccus
unc Rhodobacteraceae
Rhodobacter
unc Rhizobiales
Aurantimonas
unc Aurantimonadaceae
Methylocella
unc Beijerinckiaceae
Methylopila
unc Methylocystaceae
Afipia
Bradyrhizobium
unc Bradyrhizobiaceae
Brucella
Ochrobactrum
unc Brucellaceae
Agrobacterium
Ensifer
Rhizobium
Sinorhizobium
unc Rhizobiaceae
Ancalomicrobium
Ancylobacter
Blastochloris
Devosia
Hyphomicrobium
Labrys
Rhodoplanes
unc Hyphomicrobiaceae
Aminobacter
Aquamicrobium
Defluvibacter
Hoeflea
Mesorhizobium
Phyllobacterium
Pseudaminobacter
unc Phyllobacteriaceae
Acidithiobacillus
Alteromonas
Allochromatium
unc Gammaproteobacteria
Photobacterium
unc Vibrionaceae
Vibrio
Fluoribacter
Legionella
unc Legionellaceae
Tatlockia
Alcanivorax
unc Oceanospirillales
Halomonas
unc Halomonadaceae
Actinobacillus
Aggregatibacter
Haemophilus
Mannheimia
unc Pasteurellaceae
Enhydrobacter
unc Moraxellaceae
unc Pseudomonadales
Pseudomonas
unc Pseudomonadaceae
Hydrocarbonphaga
Lysobacter
Pseudoxanthomonas
Stenotrophomonas
Thermomonas
unc Xanthomonadaceae
Brenneria
Buchnera
Citrobacter
Dickeya
Enterobacter
Erwinia
Escherichia
Klebsiella
Kluyvera
Leclercia
Pantoea
Pectobacterium
Plesiomonas
Pragia
Salmonella
Serratia
Shigella
Xenorhabdus
unc Enterobacteriaceae
Enterobacteriales
Xanthomonadales
Pseudomonadaceae
Pseudomonadales
Pasteurellales
Halomonadaceae
Oceanospirillales
Legionellales
Vibrionales
Phyllobacteriaceae
Hyphomicrobiaceae
Rhizobiaceae
Brucellaceae
Rhizobiales
Bradyrhizobiaceae
Methylocystaceae
Beijerinckiaceae
Aurantimonadaceae
Alphaproteobacteria
Rhodobacterales
Sphingomonadales
Rickettsiales
Proteobacteria
Betaproteobacteria
Deltaproteobacteria
Burkholderiales
Burkholderiaceae
Incertae sedis 5
Comamonadaceae
Syntrophobacterales
Syntrophaceae
Desulfovibrionales
Ruminococcaceae
Lachnospiraceae
Veillonellaceae
Peptococcaceae
Peptococcaceae 2
Clostridiales
Incertae Sedis XIII
Incertae Sedis XI
Heliobacteriaceae
Clostridiaceae
Peptostreptococcaceae
Clostridia
Thermoanaerobacterales
Bacillaceae 1
Bacillaceae
Paenibacillaceae
Bacillales
Bacilli
Firmicutes
Streptococcaceae
Carnobacteriaceae
Lactobacillales
Erysipelotrichi
Porphyromonadaceae
Rikenellaceae
Prevotellaceae
Bacteroidaceae
Bacteroidetes
Flexibacteraceae
Sphingobacteria
Flavobacteria
Bacteroidetes
Propionibacterineae
Micrococcineae
Frankineae
Corynebacterineae
Actinomycetales
Actinobacteridae
Coriobacteridae
Actinobacteria
Acidobacteria
Fusobacteria
Verrucomicrobia
Nitrospira

C

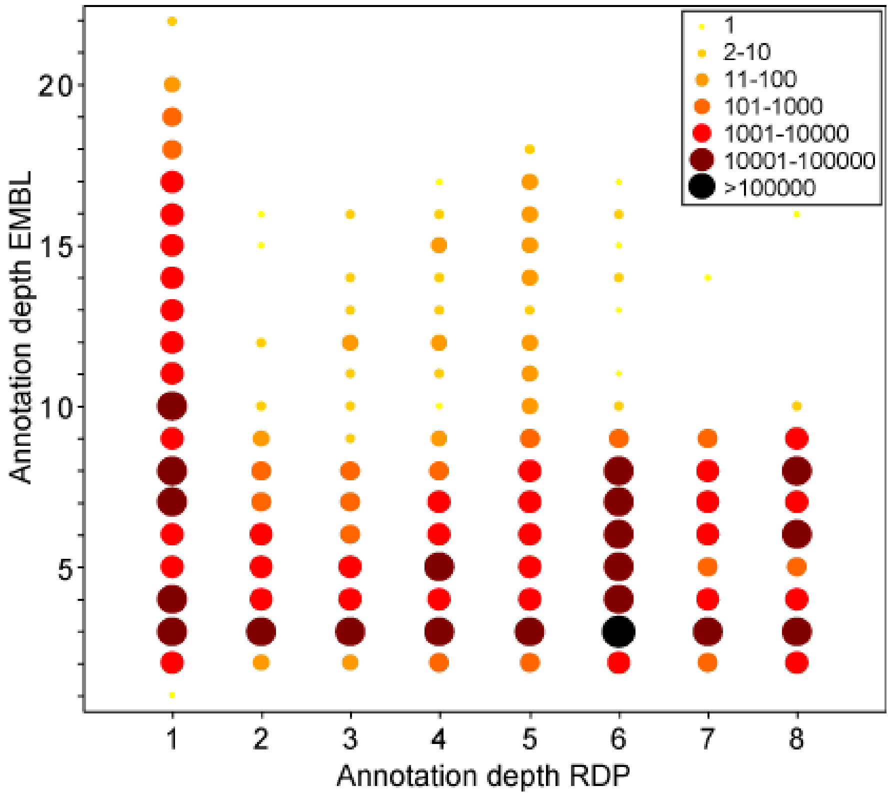

REFERENCES

1 Pruesse E, Quast C, Knittel K et al. SILVA: a comprehensive online resource for quality checked and aligned ribosomal RNA sequence data compatible with ARB. Nucl Acids Res 2007;35:7188-7196.
