# Supplementary material for: Towards the Human Colorectal Cancer Microbiome
Source: PLoS One. 2011 May 24;6(5):e20447. doi: 10.1371/journal.pone.0020447 (PMC3101260; doi:10.1371/journal.pone.0020447)
Supplement: Table S1 — Primers used in this study. (PDF) [file pone.0020447.s005.pdf]

**Table S1.** Primers used in this study.

| Primer | Reference | Sequence |
|---|---|---|
| **DGGE** | | |
| 27f | [1] | 5'-AGAGTTTGATYMTGGCTCAG |
| 1492r | [1] | 5'-TACGGYTACCTTGTTACGACTT |
| L1401r | [2] | 5'-CGGTGTGTACAAGACCC |
| 968f-GC | [2] | 5'-CCCGCCGCGCCCCGCGCCCGTCCCG CCGCCCCCGCCCGAACGCGAAGAACCTTAC |
| **RISA** | | |
| 1406f | [3] | 5′-TGYACACACCGCCCGT |
| 23Sr | [3] | 5′-GGGTTBCCCCATTCRG |
| **454 Pyrosequencing** | | |
| 16S_V1V3_MIDA_For | This study | 5′-ACGAGTGCGTGCCTAACACATGCAAGTC |
| 16S_V1V3_MIDA_Rev | This study | 5′-ACGAGTGCGTATTACCGCGGCTGCTGG |
| 16S_V1V3_MIDB_For | This study | 5′-ACGCTCGACAGCCTAACACATGCAAGTC |
| 16S_V1V3_MIDB_Rev | This study | 5′-ACGCTCGACAATTACCGCGGCTGCTGG |
| 16S_V1V3_MIDC_For | This study | 5′-AGACGCACTCGCCTAACACATGCAAGTC |
| 16S_V1V3_MIDC_Rev | This study | 5′-AGACGCACTCATTACCGCGGCTGCTGG |
| 16S_V1V3_MIDD_For | This study | 5′-AGCACTGTAGGCCTAACACATGCAAGTC |
| 16S_V1V3_MIDD_Rev | This study | 5′-AGCACTGTAGATTACCGCGGCTGCTGG |
| 16S_V1V3_MIDE_For | This study | 5′-ATCAGACACGGCCTAACACATGCAAGTC |
| 16S_V1V3_MIDE_Rev | This study | 5′-ATCAGACACGATTACCGCGGCTGCTGG |
| 16S_V1V3_MIDF_For | This study | 5′-CGTGTCTCTAGCCTAACACATGCAAGTC |
| 16S_V1V3_MIDF_Rev | This study | 5′-CGTGTCTCTAATTACCGCGGCTGCTGG |
| 16S_V1V3_MIDG_For | This study | 5′-CTCGCGTGTCGCCTAACACATGCAAGTC |
| 16S_V1V3_MIDG_Rev | This study | 5′-CTCGCGTGTCATTACCGCGGCTGCTGG |
| 16S_V1V3_MIDH_For | This study | 5′-TAGTATCAGCGCCTAACACATGCAAGTC |
| 16S_V1V3_MIDH_Rev | This study | 5′-TAGTATCAGCATTACCGCGGCTGCTGG |
| 16S_V1V3_MIDI_For | This study | 5′-TCTCTATGCGGCCTAACACATGCAAGTC |
| 16S_V1V3_MIDI_Rev | This study | 5′-TCTCTATGCGATTACCGCGGCTGCTGG |
| 16S_V1V3_MIDJ_For | This study | 5′-TGATACGTCTGCCTAACACATGCAAGTC |
| 16S_V1V3_MIDJ_Rev | This study | 5′-TGATACGTCTATTACCGCGGCTGCTGG |
| 16S_V1V3_MIDK_For | This study | 5′-TACTGAGCTAGCCTAACACATGCAAGTC |
| 16S_V1V3_MIDK_Rev | This study | 5′-TACTGAGCTAATTACCGCGGCTGCTGG |
| 16S_V1V3_MIDL_For | This study | 5′-ATATCGCGAGGCCTAACACATGCAAGTC |
| 16S_V1V3_MIDL_Rev | This study | 5′-ATATCGCGAGATTACCGCGGCTGCTGG |

1. Lane DJ et al. 16S/23S rRNA Sequencing. Nucleic Acids Techniques in Bacterial Systematics. Chichester: John Wiley & Sons, 1991:115-175.
2. Nubel U et al Sequence heterogeneities of genes encoding 16S rRNAs in Paenibacillus polymyxa detected by temperature gradient gel electrophoresis. J Bact 1996;178:5636-5643.
3. Borneman J et al. Molecular microbial diversity in soils from eastern Amazonia: Evidence for unusual microorganisms and microbial population shifts associated with deforestation. Appl Environ Microbiol 1997;63:2647-2653.
