# Supplementary material for: Towards the Human Colorectal Cancer Microbiome
Source: PLoS One. 2011 May 24;6(5):e20447. doi: 10.1371/journal.pone.0020447 (PMC3101260; doi:10.1371/journal.pone.0020447)
Supplement: Table S2 — MOTHUR diversity indices of bacterial communities in samples on- and off-tumor (Xon and Xoff, respectively). (PDF) [file pone.0020447.s006.pdf]

Table S2. MOTHUR diversity indices [1] of bacterial communities in samples on- and off-tumor (X_{on} and X_{off}, respectively).

Sample/cutoff	nseqs	sobs	ace	ace_lci	ace_hci	chao	chao_lci	chao_hci	shannon	shannon_lci	shannon_hci	simpson	simpson_lci	simpson_hci	coverage
**A_{off}**															
0.03		1128	2951.08	2814.37	3098.88	2097.49	1899.07	2346.98	5.04	5.00	5.07	0.0191	0.0184	0.0199	0.9553
0.05	12921	626	1341.99	1295.93	1391.21	1031.92	921.48	1183.64	3.68	3.65	3.72	0.0795	0.0769	0.0822	0.9780
0.10		279	515.65	461.00	586.71	415.88	362.08	504.53	2.17	2.13	2.21	0.2914	0.2851	0.2977	0.9916
**A_{on}**															
0.03		2313	5854.35	5706.74	6008.37	4155.88	3881.76	4477.91	5.64	5.61	5.66	0.0173	0.0167	0.0180	0.9528
0.05	24462	1233	2907.68	2799.21	3023.66	2173.91	1981.80	2415.30	4.44	4.41	4.47	0.0520	0.0504	0.0536	0.9765
0.10		496	909.08	834.74	999.74	751.61	671.18	868.97	3.02	3.00	3.05	0.1545	0.1508	0.1582	0.9920
**B_{off}**															
0.03		1499	4382.02	4246.81	4523.89	3085.00	2794.57	3440.53	5.80	5.77	5.83	0.0082	0.0078	0.0086	0.9394
0.05	13085	771	2092.14	1982.20	2212.05	1470.50	1299.11	1697.51	4.20	4.17	4.23	0.0427	0.0413	0.0441	0.9701
0.10		359	926.29	827.73	1045.59	641.81	545.80	787.16	2.55	2.51	2.59	0.2550	0.2475	0.2624	0.9869
**B_{on}**															
0.03		975	2233.03	2140.54	2332.86	1630.64	1482.67	1821.74	5.33	5.30	5.36	0.0103	0.0099	0.0107	0.9662
0.05	13149	429	806.32	734.24	895.41	633.95	567.63	732.00	3.63	3.60	3.66	0.0569	0.0553	0.0584	0.9865
0.10		162	278.83	242.65	331.23	228.12	195.86	291.13	1.71	1.67	1.74	0.4126	0.4031	0.4221	0.9956
**C_{off}**															
0.03		2470	6735.92	6565.57	6913.36	4522.41	4229.61	4863.94	5.99	5.97	6.02	0.0123	0.0117	0.0130	0.9425
0.05	22080	1190	2501.43	2396.20	2615.84	1921.53	1770.97	2111.11	4.57	4.54	4.60	0.0326	0.0316	0.0336	0.9757
0.10		407	546.17	503.83	607.04	542.61	492.90	621.10	3.04	3.01	3.06	0.1415	0.1384	0.1447	0.9942
**C_{on}**															
0.03		2218	6187.86	6017.08	6366.31	4258.75	3956.72	4613.24	6.19	6.16	6.22	0.0067	0.0064	0.0070	0.9150
0.05	14051	1153	2845.02	2721.74	2977.99	2039.14	1856.60	2269.04	5.02	4.99	5.06	0.0205	0.0197	0.0213	0.9602
0.10		440	937.29	846.71	1048.05	741.95	644.91	884.96	3.57	3.53	3.60	0.0737	0.0715	0.0758	0.9862
**D_{off}**															
0.03		1405	3453.26	3342.86	3569.95	2454.11	2257.28	2696.41	5.64	5.61	5.67	0.0134	0.0127	0.0142	0.9438
0.05	12146	734	2178.68	2086.93	2276.66	1411.88	1242.79	1637.18	4.58	4.55	4.61	0.0303	0.0289	0.0317	0.9690
0.10		354	932.12	829.64	1056.67	621.11	531.87	755.10	2.82	2.79	2.86	0.1904	0.1837	0.1971	0.9856
**D_{on}**															
0.03		855	1807.15	1716.57	1907.25	1429.80	1289.58	1615.26	5.16	5.14	5.19	0.0122	0.0117	0.0126	0.9700
0.05	12499	392	923.86	830.84	1036.60	746.58	627.08	926.84	3.48	3.45	3.51	0.0627	0.0612	0.0642	0.9852
0.10		136	236.56	203.85	285.04	194.33	164.42	255.75	1.39	1.36	1.42	0.5296	0.5190	0.5403	0.9960
**E_{off}**															
0.03		1556	4060.41	3929.59	4198.43	2869.96	2635.05	3156.01	5.52	5.49	5.54	0.0105	0.0102	0.0109	0.9590
0.05	19112	718	1447.16	1387.00	1512.72	1110.50	1009.77	1246.01	3.89	3.86	3.91	0.0517	0.0504	0.0530	0.9835
0.10		239	406.01	362.51	464.82	339.09	295.77	415.47	2.21	2.18	2.24	0.2371	0.2324	0.2417	0.9957
**E_{on}**															
0.03		2687	8508.43	8293.10	8732.04	5749.53	5337.71	6225.34	6.53	6.50	6.56	0.0042	0.0040	0.0044	0.8858
0.05	13589	1559	4341.31	4191.59	4499.54	3096.00	2824.80	3425.29	5.50	5.47	5.53	0.0137	0.0131	0.0143	0.9386
0.10		686	1782.97	1707.44	1864.08	1283.97	1127.29	1496.27	4.08	4.05	4.12	0.0660	0.0631	0.0689	0.9754
**F_{off}**															
0.03		3639	10277.14	10063.30	10498.09	7508.11	7050.17	8027.53	6.42	6.39	6.44	0.0079	0.0075	0.0082	0.9103
0.05	22502	1932	3965.83	3857.42	4080.34	3175.60	2969.75	3422.28	5.15	5.12	5.18	0.0300	0.0290	0.0310	0.9609
0.10		682	1278.56	1235.96	1324.43	1110.13	990.81	1275.53	3.32	3.28	3.35	0.1630	0.1586	0.1674	0.9878
**F_{on}**															
0.03		1547	4078.84	3953.77	4210.40	2997.51	2739.83	3310.85	5.44	5.41	5.47	0.0140	0.0135	0.0146	0.9432
0.05	14284	820	1631.65	1567.66	1701.11	1344.33	1215.80	1514.61	4.14	4.10	4.17	0.0538	0.0519	0.0557	0.9745
0.10		302	425.11	384.70	485.25	415.75	370.89	489.82	2.61	2.58	2.64	0.1908	0.1861	0.1955	0.9926

Nseqs: number of reads analysed, sobs: species observed at the cut-off value, ace: abundance-based coverage estimator, chao: abundance-based estimator Chao1, Shannon: Shannon index of diversity, Simpson: Simpson index of diversity, Coverage: Good's coverage. LCI and HCI: lower and higher confidence interval, respectively.

1. Schloss PD et al Introducing mothur: Open Source, Platform-independent, Community-supported Software for Describing and Comparing Microbial Communities. Appl Environ Microbiol 2009;75:7537-7541.
