# Supplementary material for: Towards the Human Colorectal Cancer Microbiome
Source: PLoS One. 2011 May 24;6(5):e20447. doi: 10.1371/journal.pone.0020447 (PMC3101260; doi:10.1371/journal.pone.0020447)
Supplement: Table S3 — Genus level comparison generated with the RDP library compare tool. Values indicate the number of 16S rRNA pyrosequencing reads that map to the listed genus. Only significant differences are shown (P<0.05). A–F: patients; Xoff: off-tumor tissue; Xon: on-tumor tissue. (PDF) [file pone.0020447.s007.pdf]

Table S3. Genus level comparison generated with the RDP library compare tool [1]. Values indicate the number of 16S rRNA pyrosequencing reads that map to the listed genus. Only significant differences are shown (P < 0.05). A-F: patients; X_{off}: off-tumor tissue; X_{on}: on-tumor tissue.

A_{off} vs A_{on}	A_{off}	A_{on}	P value
**Megamonas**	406	1	<0.0001
**Subdoligranulum**	5	179	<0.0001
**Granulicatella**	59	2	<0.0001
**Coprobacillus**	54	4	<0.0001
**"Peptostreptococcaceae Incertae Sedis"**	44	11390	<0.0001
**Anaerobacter**	96	16	<0.0001
**Bacteroides**	249	4778	<0.0001
**Clostridium**	2971	980	<0.0001
**Parabacteroides**	36	515	<0.0001
**Sutterella**	6559	13	<0.0001
**"Ruminococcaceae Incertae Sedis"**	9	143	<0.0001
**Collinsella**	37	239	<0.0001
**Faecalibacterium**	84	356	<0.0001
**Eggerthella**	0	53	<0.0001
**Veillonella**	1	49	<0.0001
**"Lachnospiraceae Incertae Sedis"**	171	521	<0.0001
**Bacillus c**	13	0	<0.0001
**Shigella**	27	16	<0.0001
**Haemophilus**	8	0	<0.0001
**Propionibacterium**	8	0	<0.0001
**Gemella**	10	2	0.001
**Peptostreptococcus**	0	17	0.001
**Anaerovorax**	4	0	0.010
**Flavimonas**	4	0	0.010
**Enterococcus**	3	0	0.029
**Anaerotruncus**	0	9	0.029
**Alistipes**	0	8	0.044

B_{off} vs B_{on}	B_{off}	B_{on}	P value
**Porphyromonas**	950	0	<0.0001
**Moryella**	166	0	<0.0001
**Dialister**	153	0	<0.0001
**Rothia**	69	0	<0.0001
**Gemella**	68	0	<0.0001
**Prevotella**	1	59	<0.0001
**"Peptostreptococcaceae Incertae Sedis"**	426	165	<0.0001
**Bacteroides**	296	1192	<0.0001
**Escherichia**	798	52	<0.0001
**Faecalibacterium**	165	988	<0.0001
**Parvimonas**	621	101	<0.0001
**Peptostreptococcus**	2206	6	<0.0001
**Shigella**	776	2399	<0.0001
**"Lachnospiraceae Incertae Sedis"**	20	80	<0.0001
**Anaerovorax**	28	0	<0.0001
**Bulleidia**	16	0	<0.0001
**Fusobacterium**	0	14	<0.0001
**Paracoccus**	0	14	<0.0001
**Collinsella**	1	13	0.001
**Coprobacillus**	0	10	0.001
**TM7_genera_incertae_sedis**	10	0	0.001
**Propionibacterium**	9	0	0.002
**Actinomyces**	10	1	0.007
**Granulicatella**	11	2	0.013
**Staphylococcus**	5	0	0.032

C_{off} vs C_{on}	C_{off}	C_{on}	P value
"Peptostreptococcaceae Incertae Sedis"	0	2892	<0.0001
Rothia	0	741	<0.0001
Granulicatella	0	513	<0.0001
Gemella	0	336	<0.0001
Sphingomonas	466	0	<0.0001
Mogibacterium	0	238	<0.0001
Veillonella	0	200	<0.0001
Sutterella	0	127	<0.0001
Peptostreptococcus	0	108	<0.0001
Enhydrobacter	169	0	<0.0001
Clostridium	154	0	<0.0001
Parvimonas	0	58	<0.0001
TM7_genera_incertae_sedis	0	55	<0.0001
Actinomyces	0	41	<0.0001
Bacteroides	2531	35	<0.0001
Haemophilus	122	1585	<0.0001
Propionibacterium	5856	45	<0.0001
Shigella	1062	366	<0.0001
Corynebacterium	62	0	<0.0001
Subdoligranulum	0	27	<0.0001
Staphylococcus	296	89	<0.0001
Bulleidia	0	20	<0.0001
Actinobacillus	0	17	<0.0001
Nesterenkonia	0	17	<0.0001
Prevotella	0	17	<0.0001
Dialister	0	15	<0.0001
Paenibacillus	20	0	<0.0001
Phocoenobacter	6	18	<0.0001
Porphyromonas	51	63	<0.0001
"Lachnospiraceae Incertae Sedis"	0	8	<0.0001
Anaerobacter	19	1	0.001
Lactobacillus	0	6	0.003
Neisseria	11	20	0.003
Bacillus c	24	4	0.006
Jeotgalicoccus	0	5	0.007
Parabacteroides	0	5	0.007
Streptococcus	5	11	0.016
Moryella	0	4	0.018

D_{off} vs D_{on}	D_{off}	D_{on}	P value
**"Lachnospiraceae Incertae Sedis"**	195	572	<0.0001
**"Peptostreptococcaceae Incertae Sedis"**	1015	53	<0.0001
**Propionibacterium**	94	0	<0.0001
**Anaerovorax**	50	0	<0.0001
**Granulicatella**	44	0	<0.0001
**Bacteroides**	802	1379	<0.0001
**Faecalibacterium**	105	359	<0.0001
**Subdoligranulum**	812	155	<0.0001
**Eggerthella**	38	0	<0.0001
**Sutterella**	38	0	<0.0001
**Clostridium**	36	0	<0.0001
**Haemophilus**	25	0	<0.0001
**TM7_genera_incertae_sedis**	24	0	<0.0001
**Peptostreptococcus**	20	0	<0.0001
**Dialister**	19	0	<0.0001
**Enterococcus**	12	45	<0.0001
**Shigella**	1082	1286	0.001
**Desulfovibrio**	9	0	0.002
**Prevotella**	9	0	0.002
**Parabacteroides**	8	0	0.003
**Staphylococcus**	10	1	0.005
**Parvimonas**	7	0	0.007
**Escherichia**	11	2	0.011
**Enhydrobacter**	5	0	0.028

E_{off} vs E_{on}	E_{off}	E_{on}	P value
**Haemophilus**	0	555	<0.0001
**Propionibacterium**	0	464	<0.0001
**Sutterella**	0	449	<0.0001
**Bradyrhizobium**	0	334	<0.0001
**Rothia**	1	238	<0.0001
**Parabacteroides**	0	152	<0.0001
**Acinetobacter**	0	149	<0.0001
**Veillonella**	0	149	<0.0001
**Capnocytophaga**	0	124	<0.0001
**Alistipes**	1	128	<0.0001
**Prevotella**	0	113	<0.0001
**Paracoccus**	0	92	<0.0001
**Campylobacter**	0	75	<0.0001
**Staphylococcus**	0	60	<0.0001
**Anaerococcus**	0	48	<0.0001
**Phyllobacterium**	0	46	<0.0001
**Granulicatella**	0	40	<0.0001
**TM7_genera_incertae_sedis**	0	40	<0.0001
**Bulleidia**	0	39	<0.0001
**Aggregatibacter**	0	38	<0.0001
**"Lachnospiraceae Incertae Sedis"**	692	802	<0.0001
**"Peptostreptococcaceae Incertae Sedis"**	75	205	<0.0001
**Bacteroides**	4123	547	<0.0001
**Collinsella**	18	114	<0.0001
**Escherichia**	6	495	<0.0001
**Mesorhizobium**	0	34	<0.0001
**Finegoldia**	0	30	<0.0001
**Flavimonas**	0	29	<0.0001
**Fusobacterium**	0	25	<0.0001
**Porphyromonas**	0	25	<0.0001
**Gemella**	0	21	<0.0001
**Ruminococcus**	0	21	<0.0001
**Subdoligranulum**	147	39	<0.0001
**Roseburia**	5	30	<0.0001
**Pseudomonas**	0	17	<0.0001
**Dermacoccus**	0	14	<0.0001
**Faecalibacterium**	823	729	<0.0001
**Dialister**	0	11	<0.0001
**Neisseria**	0	11	<0.0001
**Actinomyces**	0	10	<0.0001
**Modestobacter**	0	8	0.001
**Coprococcus**	12	0	0.002
**Enterococcus**	12	0	0.002
**Corynebacterium**	0	6	0.004
**Xylanibacter**	0	4	0.025

F_{off} vs F_{on}	F_{off}	F_{on}	P value
**"Ruminococcaceae Incertae Sedis"**	1260	0	<0.0001
**Butyrivibrio**	752	0	<0.0001
**Prevotella**	0	134	<0.0001
**Coprobacillus**	0	77	<0.0001
**Collinsella**	1	63	<0.0001
**Parvimonas**	0	37	<0.0001
**"Lachnospiraceae Incertae Sedis"**	171	340	<0.0001
**"Peptostreptococcaceae Incertae Sedis"**	10	413	<0.0001
**Bacteroides**	319	3980	<0.0001
**Coprococcus**	7	98	<0.0001
**Faecalibacterium**	111	6055	<0.0001
**Shigella**	77	1005	<0.0001
**Streptococcus**	55	2	<0.0001
**Erysipelotrichaceae Incertae Sedis**	0	22	<0.0001
**Peptostreptococcus**	0	18	<0.0001
**Alistipes**	35	0	<0.0001
**Selenomonas**	35	0	<0.0001
**Veillonella**	8	33	<0.0001
**Dorea**	1	14	<0.0001
**Sutterella**	15	32	<0.0001
**Bradyrhizobium**	12	0	0.003
**Actinomyces**	1	6	0.014
**Anaerovorax**	9	0	0.014
**Xylanibacter**	9	0	0.014
**Rothia**	11	1	0.031

1. Lane DJ et al. 16S/23S rRNA Sequencing. Nucleic Acids Techniques in Bacterial Systematics. Chichester: John Wiley & Sons, 1991:115-175.
